# Dichotomous keys for morphological identification of spores produced by arbuscular mycorrhizal fungi (phylum Glomeromycota) integrating the mode of spore formation and a spore developmental model

**DOI:** 10.1007/s00572-026-01270-7

**Published:** 2026-06-20

**Authors:** Sidney L. Stürmer, Karl Kemmelmeier, Terra K. Lubin, Liz Koziol, Peggy A. Schultz, James D. Bever, Joseph B. Morton

**Affiliations:** 1 Departamento de Ciências Naturais, Universidade Regional de Blumenau, Blumenau, SC 89030-903 Brazil; 2 Kansas Biological Survey, Department of Ecology and Evolution, University of Kansas, Lawrence, KS 66047 USA; 3 Plant and Soil Sciences, West Virginia University, Morgantown, WV 26506 USA

**Keywords:** Ontogeny, Taxonomy, Species identification, Spore morphology, Spore traits

## Abstract

Taxonomy of arbuscular mycorrhizal fungi (AMF) fungi (Glomeromycota) has long relied on spore morphological characteristics, which are distinguishable and evolutionarily stable structures in these fungi. Mode of spore formation and germination properties in AMF identify higher order taxa. Species level traits include external and internal spore morphology (*e.g.* size, color, spore wall structure, surface ornamentation) and some characteristics at the region of hyphal attachment. Comparative studies of AMF spore ontogeny led to a developmental model where characters were defined by their origin, position in ontogeny, and constraints on variation. The goals of this work are (i) to revisit the spore developmental model, the model’s premises and interpretations for morphological taxonomy of AMF, (ii) to describe and compare the main modes of spore formation as the first step to define taxa, and (iii) to organize these data into dichotomous keys as a tool for AMF species identification. Such an aid will facilitate a more accurate identification of fungal specimens collected and investigated in field-based and ecological studies, in organization and maintenance of germplasm deposited in culture collections, and in production and utilization of commercial inoculants.

**Supplementary Information:**

The online version contains supplementary material available at 10.1007/s00572-026-01270-7.

## Introduction

Arbuscular mycorrhizal fungi (AMF) comprise a group of *ca.* 355 species of soil fungi that establish the arbuscular mycorrhizal symbiosis with roots of a wide range of plant families (Wang and Qiu 2006; Brundrett and Tedersoo 2018). Fossilized spores place the origin of AMF 460 million years ago at the Ordovician (Redecker et al. 2000) and evidence from the SSU rRNA gene (Davison et al. 2015), population genetics (Rosendahl et al. 2009; Savary et al. 2018), and field-collected spores (Stürmer et al. 2018) reveal that some species are cosmopolitan, being detected in agricultural fields and pristine ecosystems, across different continents under distinct climatic and soil conditions. In association with plant roots, AMF differentiate arbuscules and interconnecting internal mycelium within the root cortex, and form an extensive extraradical mycelium network that acquire poorly soluble nutrients in soil and produce extraradical and/or intraradical resting spores in the rhizosphere (Smith and Read 2008).

A distinct feature of AMF is that their spores are multinucleate and among the largest known within the kingdom Fungi (Aguilar-Trigueros et al. 2019), with a diameter range of 25 to 800 µm. The spores serve as propagules to initiate root colonization, are capable of long-distance dispersal (Koske and Gemma 1990; Chaudhary et al. 2020) and bear traits to resist stressful conditions (Deveautour et al. 2020; Hopkins and Bennett 2023). As with other fungi, AMF spores vary in color, shape, wall thickness, and other external features such as surface ornamentation or protuberances, and are formed singly, in loose clusters or in sporocarps (Morton 1988; Walker 1983). Many taxa form spores much like other fungi from a single hypha. Other taxa form AMF spores in unique ways with precursor structures or are dimorphic with divergent modes of formation.

Among the different structures differentiated by AMF, only spores have discrete divergent phenotypes that can group fungi from different populations and geographical locations into a species (Morton 1993). What really sets spores of many AMF species apart from other fungal taxa is the range and complexity of inner morphological components. Those associated with the spore wall may be simple or consist of multiple discrete layers that often can be visualized with staining methods. However, many AMF species form within each spore additional colorless flexible layers of varying thickness and plasticity as a prelude to unique germination events.

Since the first description of *Glomus macrocarpum* by Tulasne and Tulasne in 1845, erection of new taxa and description of new species have been based mainly on the morphological features of their spores. Early species descriptions lacked a standardized terminology for the types and range of structures that embody spore phenotypes, which possibly accounts for the inaccurate or incomplete species description, including redescription of some taxa (Morton 1993). Walker (1983) was the first to propose a standardization of terminology differentiating stable phenotypes present in AMF spores. He referred to any distinct layers forming the spore as “walls” which could be grouped into “wall groups” according to how they separated when spores were broken and mounted on slides. Walker (1983) also introduced a “murograph” to graphically represent the different wall types and groups. He termed these walls as unit, laminated, evanescent, and membranous. Additional wall types were proposed (*e.g.* amorphous, expanding, coriaceous) as new species were described. Walker’s treatise represented an important contribution to the taxonomy of AMF as new species afterwards were described in a standard way that allowed direct comparison across taxa. Berch (1986) argued that “wall layers” instead of “wall” should be used to refer to the distinct types of spore wall components based on the origin of each structure, pointing to the value of investigating spore wall development.

Spore ontogeny was studied for some species like *Funneliformis coronatum* (as *Glomus coronatum*, Giovannetti et al. 1991), *F. mosseae* (as *Glomus mosseae*, Meier and Charvat 1992), and *Paracorymbiglomus globiferum* (as *Glomus globiferum*, Wu and Sylvia 1993), but they did not focus on the differentiation of spore subcellular structures and its implication to resolve species (Stürmer and Morton 1997) nor did they compare geographical isolates of the same species to determine stability of characters during spore ontogeny. Given the taxonomic range and morphological diversity of phenotypes housed at INVAM (International Culture Collection of (Vesicular) Arbuscular Mycorrhizal Fungi), a major research focus was reinterpreting AMF spore subcellular structures based on their origin and developmental patterns (Stürmer et al. 2021). The output of several comparative studies culminated in a spore developmental model proposed by Morton et al. (1995) that was applicable to all families recognized at that time and a proposed nomenclature for spore phenotypes based on ontogenetic boundaries between characters.

Systematics of Glomeromycota has changed radically since the early 2000 s as genetic characters were introduced to elucidate phylogenetic relationships between clades. New species descriptions based on spore morphology usually includes a gene phylogeny (*e.g.* SSU or LSU rRNA, *rpb*1) to demonstrate phylogenetic relationships with sister taxa. As a consequence, reference data sets based on the SSU rRNA gene (Öpik et al. 2010), the SSU-ITS-LSU rDNA region (Krüger et al. 2012), and the LSU rRNA gene (Delavaux et al. 2021, 2022) were developed for molecular systematic and community studies. Erection of new species and genera based on environmental DNA (eDNA) (Tedersoo et al. 2024) and implementation of genome-based approaches for species classification (Corradi et al. 2025) have been proposed. While the quest to standardize species-, genus- or family-level identification and classification within Glomeromycota using molecular methods continues, the species concept for AMF centered on the morphology and organizational properties of their spores, as conceptualized by Morton et al. (1992), remains the standard for the discipline. Spore morphology remains the most accessible and often the first-line approach for AMF identification even as SSU- or LSU- based metabarcoding became the standard method for profiling AMF communities.

To accurately identify AMF species based on morphology, researchers can utilize dichotomous keys that presents a series of choices that lead the users step-by-step to the correct taxon. Gerdemann and Trappe (1974) were the first to introduce keys for all known species in *Glomus, Acaulospora,* and *Gigaspora.* Subsequent taxonomic papers by N.C. Schenck (*e.g.* Nicolson and Schenck 1979; Schenck and Smith 1982; Schenck et al. 1984), R.E. Koske and C. Walker (*e.g.* Koske and Walker 1985) included synoptic or dichotomous key for species within genera or group of species with particular traits. In the last decade, with the proliferation of new taxa in all taxonomic ranks, many publications also included keys for identification of species within genera (*e.g.* Oehl et al. 2012; Palenzuela et al. 2013; Corazon-Guivin et al. 2022) or genera (Silva et al. 2023). Some keys reported in the literature are useful for a given group of taxa while others are quite cumbersome, require substantial taxonomic expertise and therefore not user friendly. Therefore, a dichotomous key which considers the mode of spore formation, a standard nomenclature for spore phenotypes and which encompases most of the species in Glomeromycota is needed. Such taxonomic keys are an important tool for proper identification of AMF found in ecological studies, biodiversity assessments, culture collections, and commercial inoculants.

AMF taxonomy still relies strongly on the morphological analysis of their large multinucleate spores (Morton 1988; Walker and Vestberg 1998), with an increasing rate of description of new species of AMF in recent years and, perhaps more importantly, a renewed focus on spore-based studies to elucidate functional significance of spore traits (Deveautour et al. 2020; Hopkins and Bennett 2023; Chaudhary et al. 2025). To assist in those efforts, we aimed in this paper (i) to revisit the spore developmental model proposed by Morton et al. (1995), its premises and implications for morphological taxonomy of AMF, (ii) to clarify comparative details of the main modes of spore formation as the first step in defining taxonomic location of a species, and (iii) to provide dichotomous keys for identification of AMF at the species level.

## The spore development model for AMF in a nutshell

**Fig. 1:**
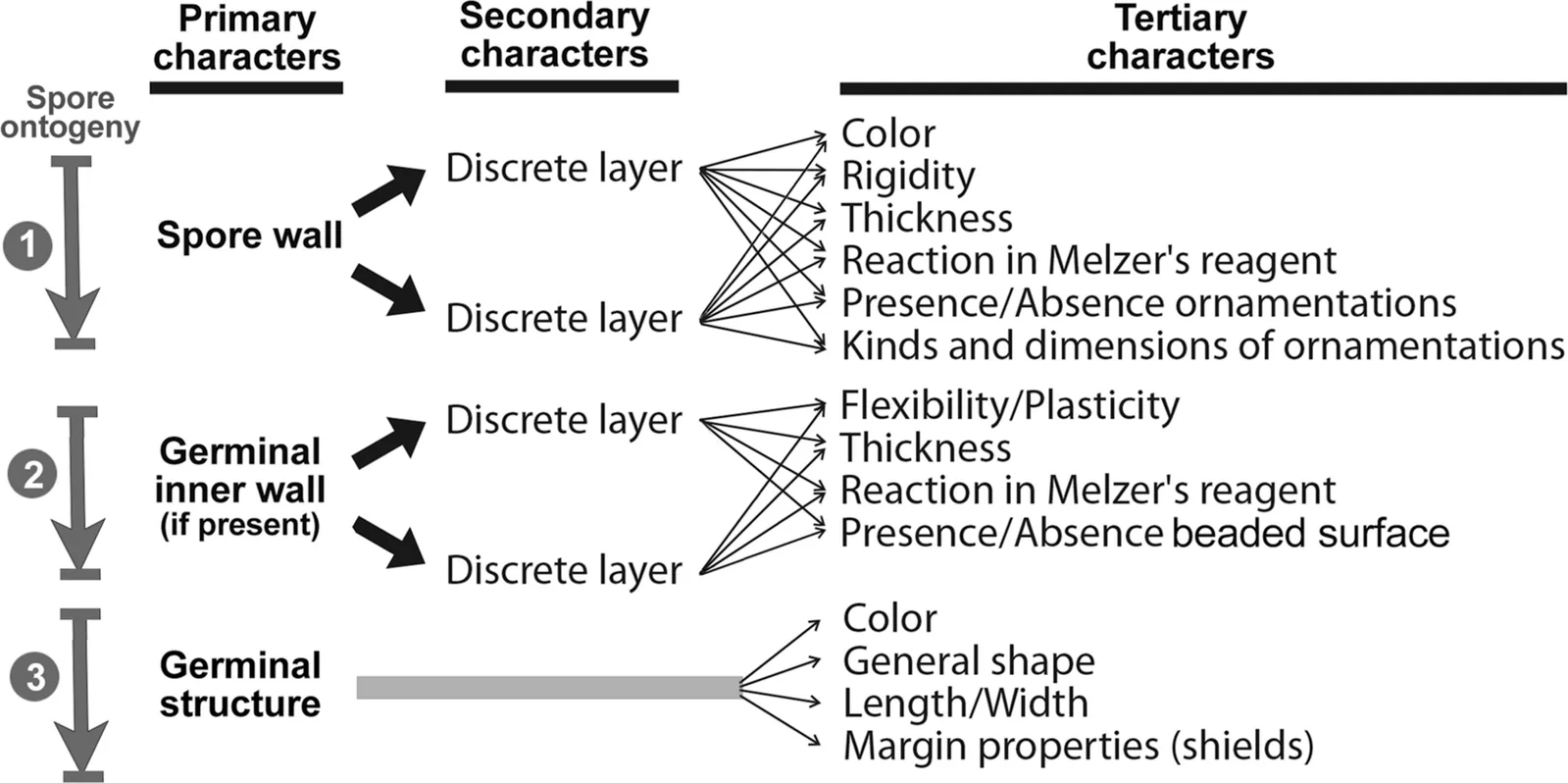
A developmental model depicting the discrete stages in the formation and differentiation of phenotypic characters in spores that inform on morphological evolution of glomeromycotan fungi. Numbered vertical arrows denote the sequential stages in formation of primary characters wherein each character completes differentiation before the next stage is initiated. Secondary characters are layers (components) differentiated within primary characters. Layers are sufficiently constrained to group species into higher taxa and their number ranges from 1 to 6 depending on the species (only 2 layers depicted herein for simplicity). Tertiary characters are expressed in each secondary character. They exhibit few constraints and therefore account for most species-level variation

The study of development provides insights not only into the results of ontogeny, but also the order in which structural characters are synthesized and interact with each other either independently or together. Morton et al. (1995) proposed a model (Fig. 1) integrating all morphological components of AMF spores based on comparative studies of species in three families recognized at that time: Gigasporaceae, including *Dentiscutata heterogama* and *Cetraspora pellucida* (as *Scutellospora heterogama* and *S. pellucida,* respectively) (Franke and Morton 1994), five species of *Racocetra* that shared one bilayered germinal wall (as *Scutellospora*) (Morton 1995), and five species of *Gigaspora* (Bentivenga and Morton 1995); Glomeraceae (Morton 1996; Stürmer and Morton 1997), and Acaulosporaceae (Stürmer and Morton 1999; Schultz et al. 1999).

All AMF spores form on a sporogenous hypha but the structures and patterns of that process vary considerably at the family and genus levels, as well as within a few species. The spore wall enclosing contents of cytoplasm and nuclear conglomerate differentiates into a single or multiple layers. Spore wall differentiation occurs during expansion to its final size and this stage is applicable to all species. For some taxa (*e.g.* species in Glomeraceae, *Gigaspora* spp.), the differentiated spore wall is the immediate precursor for germination. In other taxa, new flexible colorless structures (= inner walls) are synthesized internally following spore wall maturation. They form separately, and if there are more than one, they arise in a discrete linear sequence. Germination, the final step, occurs only after all of the inner walls have differentiated their mature phenotypes. In species differentiating germinal walls, germination appears to be linked to these inner walls (it does not occur without them) and hence they were reconsidered as *germinal walls*.

Given that all observable characters of a spore develop hierarchically in a linear order, morphology can be organized as primary, secondary, and tertiary components (Fig. 1). Primary characters are those easily recognized when spores are crushed and observed under a compound microscope: the *spore wall*, *germinal walls* (= *inner walls*), and *germination structure* (when present). As differentiation of primary characters is ordered, the onset on development of one character starts only after the offset of the previous one: differentiation of germinal walls starts only after the spore wall was completely formed and formation of germination structure occurs only after germinal walls are completely differentiated.

Secondary characters are phenotypically distinct components that form within a primary character. These characters are layers that differentiate within the spore wall and germinal walls. Number of layers varies from 1 to 6 in the spore wall and 1 to 3 in germinal walls.

Tertiary characters are qualitative and quantitative properties of individual differentiated layers forming the spore and germinal walls, and qualitative and quantitative properties of the germination structure. They include spore diameter (determined by spore wall expansion), color, rigidity, thickness, type and dimension of ornamentation, and Melzer’s reaction of layers comprising the spore wall. Tertiary characters of germinal walls include color, ornamentation, as well as plasticity, thickness, and Melzer’s reaction, the last three being causally linked (Morton 1988). Properties of the *germination structure* include color, shape (*e.g.* violin-shaped to cardioid), length/width, and margin properties (*e.g.* number of lobes or compartments) and it applies only to those genera that differentiate germination shields and orbs.

Since spore development is hierarchic, constraints on variation in primary characters is high, which is why they generally define taxa above genus. Secondary characters other than the spore wall helps to define genera in most taxa. Tertiary characters are least constrained and therefore express species-level variation for currently known species. It is important to notice that some tertiary characters can be highly variable and challenging to score consistently, particularly for non-experimented taxonomists. For instance, rigidity of a discrete layer is not easily assessed for non-experimented taxonomists and determination of color can be influenced by the observer perception or microscope settings.

The spore development model establishes character states of all layers in the *spore wall* as the main focus to be analyzed when identifying spores to the species level. Properties such as spore shape, color and size (because of spore wall expansion) are readily described under a dissecting microscope. Once spores are mounted in slides and broken, examination of secondary and tertiary *spore wall* characters under a compound microscope can be done (*e.g.*, number of layers, thicknesses, presence and pattern of ornamentation, Melzer’s reaction, etc.). *Germinal walls* (when present) will separate with applied pressure to the cover slip and their secondary and tertiary properties (*e.g.* layer number, thickness, plasticity, Melzer’s reaction, etc.) can provide information that aid discrimination of family and genus. This implies that focus during species identification of AMF shifts from analyzing all the subcellular structures (*e.g.*, thickness of layers forming a germinal wall) of a spore (some of which are hard to differentiate) to determining the properties of *spore wall* layers which are usually easier to visualize and more robust when compared to *germinal walls.* Focusing on the spore wall characters (secondary and tertiary) states facilitate beginning and experienced researchers to identify an AMF species with a reasonable degree of confidence.

A second implication of the model is that the terminology proposed (spore wall, germinal wall, germination structure, layers) to define and interpret primary and secondary characters is based on the biological process of ontogeny. Terminology pertaining to the developmental model has been accepted by some taxonomists and adopted in species descriptions (Kaonongbua et al. 2010; Symanczik et al. 2014; Błaszkowski et al. 2015). Comparative studies clearly indicate that the ontogenetic trajectory of the *spore wall* is independent of the *germinal walls* and differentiation of germination structures associated with them (Morton and Msiska 2010b). In this study, spore ontogeny of a wild type strain of *Dentiscutata heterogama* was compared to that of an albino mutant. Both possessed unique divergent phenotypes only in the tertiary characters of the spore wall caused by neoteny, wherein the mutant stopped spore wall differentiation before all aspects of the phenotype were expressed relative to the wild type. The differentiation of germinal walls and germination shield was unaffected. This result emphasizes the independence of spore wall variation from all subsequent developmental events and accounts for range of species-level variation in Glomeromycota (Morton et al. 1995).

## Modes of spore formation

**Fig. 2:**
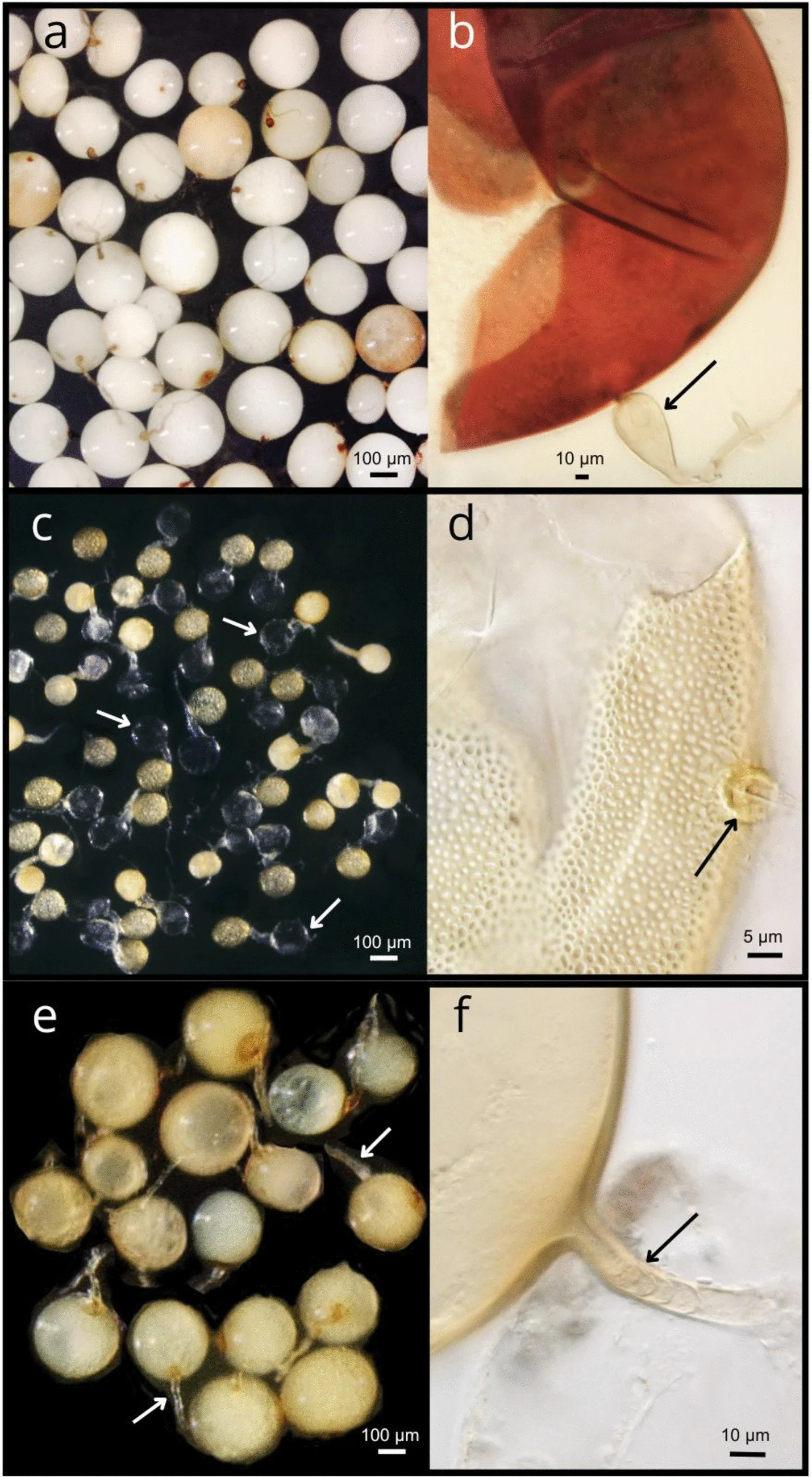
Mode of spore formation as observed under a dissecting microscope (first column) and a compound microscope (second column): **a**) gigasporoid spores of *Gigaspora margarita.* **b**) gigasporoid spore of *G. margarita* showing the bulbous suspensor-like cell (arrow). **c**) acaulosporoid spores of *Acaulospora cavernata* with sporiferous saccules (arrows) attached to spores. **d**) acaulosporoid spore of *A. cavernata* showing the cicatrix (arrow) left after the saccule detached. **e**) glomoid spores of *Funneliformis mosseae* showing the subtending hypha (arrows). **f**) glomoid spore of *Funneliformis fragilistratum* showing the subtending hypha (arrow)

The first step in AMF species identification is to recognize the mode of spore formation under the dissecting microscope as it establishes membership in the order, family, and genera of the specimen (Table 1). Modes of spore formation in AMF are patterns defined by where and how the spores form in relation to a sporogenous hypha. Three major modes of spore formation are recognized according to Walker et al. (2018): gigasporoid, acaulosporoid, and glomoid (Fig. 2).

**Table 1:** Modes of spore formation and distribution through the phylogeny in Glomeromycota

Order	Family	Genera
*Gigasporoid*: Spores differentiated at the tip of a bulbous suspensor-like cell		
Diversisporales	Gigasporaceae	*Gigaspora, Racocetra, Cetraspora, Dentiscutata, Scutellospora, Paradentiscutata, Intraornatospora, Bulbospora, Fuscutata*
*Acaulosporoid*: Formation of a sporiferous saccule and spores differentiated laterally or inside the saccule neck or inside the saccule		
Diversisporales	Acaulosporaceae	*Acaulospora, Kuklospora*
	Diversisporaceae	*Diversispora*
	Sacculosporaceae	*Sacculospora*
Entrophosporales	Entrophosporaceae	*Entrophospora*
Archaeosporales	Archaeosporaceae	*Archaeospora, Andinospora, Antiquispora*
	Ambisporaceae	*Ambispora*
	Polonosporaceae	*Polonospora*
*Glomoid*: Spores formed blastically at the tip of a sporogenous hypha or as intercalary inflation of a hypha		
Diversisporales	Diversisporaceae	*Corymbiglomus, Paracorymbiglomus, Desertispora, Diversispora, Redeckera, Sieverdingia*
	Pacisporaceae	*Pacispora*
Glomerales	Glomeraceae	*Complexispora, Sclerocarpum, Glomus*
	Dominikiaceae	*Nanoglomus, Macrodominikia, Orientoglomus, Microdominikia, Dominikia, Delicatispora*
	Septoglomeraceae	*Blaszkowskia, Funneliformis, Funneliglomus, Melanoglomus, Microviscospora, Septoglomus, Viscospora*
	Kamienskiaceae	*Microkamienskia, Kamienskia, Epigeocarpum*
	Sclerocystaceae	*Silvaspora, Sclerocystis, Rhizophagus/Rhizoglomus, Oehlia, Halonatospora*
Entrophosporales	Entrophosporaceae	*Entrophospora*
Archaeosporales	Ambisporaceae	*Ambispora*
Paraglomerales	Paraglomeraceae	*Paraglomus, Innospora*
	Pervetustaceae	*Pervetustus*

Gigasporoid spores (Fig. 2a, b) are so named in reference to the genus *Gigaspora*, the first genus described with this mode of spore formation (Gerdemann and Trappe 1974). Gigasporoid spores are borne on a bulbous suspensor-like cell differentiated at the tip of the sporogenous hypha, with one or more peg-like projections arising from the bulbous cell and branching towards the spore (Walker and Sanders 1986). One to several septa may be present in the sporogenous hypha located at some distance from the bottom of the bulbous suspensor cell. Gigasporoid spores also are mostly globose to subglobose, with other variations in few species. This mode of formation is restricted to the family Gigasporaceae in the order Diversisporales (Table 1).

Acaulosporoid spores (Fig. 2c, 2 d) are so named in reference to the genus *Acaulospora*, the largest genus with this mode of spore formation. The fungus first forms blastically a swollen balloon-like structure from a sporogenous hypha, called a sporiferous saccule. The saccule has condensed, homogenous, granular contents that extrude as a viscous stream when broken (Mosse 1970). The saccule functions as a precursor to formation of a spore and facilitates the transfer of cytoplasmic contents from the parent hypha into the developing spore (Mosse 1970). Spores are then formed (i) laterally on the saccule neck, (ii) inside the saccule neck or (iii) within the sporiferous saccule. Except for spores formed within the sporiferous saccule (only by *Acaulospora intravesiculata* so far), the saccule detaches once the spore wall is differentiated (Stürmer and Morton 1999). Remnants of the saccule might be present attached to the spore, especially if the walls of the saccule’s neck are slightly pigmented and thick. The areas of contact between the spore and the saccule’s neck leaves one or two scars that are typical of this mode of spore formation. In some species with acaulosporoid mode of formation, spores are formed at the tip of a glomoid-like sporogeneous hypha, the pedicel, that branches from the neck of the sporiferous saccule as in *Ambispora* (Bills and Morton 2015) and in few species of *Acaulospora* like *Acaulospora gedanensis* (Niezgoda et al. 2024) and *Acaulospora brasiliensis* (Krüger et al. 2011). Species with this mode of spore formation form spores mostly globose to subglobose, although a few species can form spores with other shapes. Three orders and seven families within Glomeromycota present the acaulosporoid mode of spore formation (Table 1).

Glomoid spores (Fig. 2e, 2f) bear their name from *Glomus*, the largest defining genus for the phylum Glomeromycota. Glomoid spores are formed as an inflation at the tip of a sporogenous hypha (described as blastically) or at the middle of an undifferentiated hypha (described as intercalary) (Walker et al. 2018). The wall of the sporogenous hypha serves as the origin of the spore wall and hence is continuous. Additional layers may form distally or proximally during spore differentiation. At maturity, the spore lumen is closed off by a thin membrane, hyphal wall thickening, a septum or a plug (Morton 1988; Walker et al. 2018). The widest range of variation in spore shapes is found within glomoid spores as they are mostly globose to subglobose, but also can be a variety of other shapes (see Supplementary Material S1). Shape of subtending hyphae is highly variable among species but most have a cylindric subtending hypha to flared hypha (Morton 1988). Other shapes of subtending hyphae include funnel-shaped, constricted, irregular, recurved, and bill-shaped (Morton 1988; Oehl et al. 2011). This mode of spore formation is the most common within Glomeromycota, being present in all five orders, 11 families and detected even in species of *Sclerocystis, Sclerocarpum, Epigeocarpum,* and *Redeckera* that form large and compact sporocarps. The order Glomerales and Paraglomerales are known to produce only glomoid spores (Table 1).

## Taxonomic Keys

Morphological characters of 310 species of AM fungi were obtained from the original protologue of species descriptions, website of the International Collection of (Vesicular) Arbuscular Mycorrhizal Fungi (INVAM at http://invam.ku.edu), Błaszkowski (2012), and published taxonomic papers of certain species (including updates and redescriptions of taxa). For some species, data were obtained from two different sources when they complemented each other. Data were obtained manually from the sources and separated for species forming gigasporoid, acaulosporoid, and glomoid spores (Supplementary Material S1). Characters included those that are observed under a dissecting microscope (*e.g.* spore formation singly in the soil or sporocarps, spore color, shape, and size) and those observed after spores are mounted on slides and observed under a compound microscope (*e.g.* ornamentation, number of spore wall layers, number of germinal walls, number and thickness of layers forming the spore wall, and Melzer’s reaction) (see Glossary – Supplementary Material S2). Data for spore size and thickness of layers forming the spore wall are reported as minimum, maximum, and mean values; if no mean values were reported, the midpoint of the maximum and minimum was calculated.

The total number of AM fungal species now validly described is 355 according to the list at www.amf-phylogeny.com (compiled by Dr. Arthur Schüßler and currently maintained at CICG: https://sites.google.com/view/cicg-furb-english/taxonomy/amf-species-list), but only 310 species are included in the taxonomic keys. Listing a species in the key does not automatically mean that we accept the species (as some species could be synonyms of each other). Moreover, some listed species may also be doubtful in their taxonomic position (due to lack of molecular information) and their usage is not endorsed by the present study. For species in *Glomus,* we included only those that have some molecular markers that strongly placing them in this genus. Therefore, most of species listed as of uncertain position in *Glomus *sensu lato by Schüßler and Walker (2010) were not included in the taxonomic keys, except if they were transferred to another genus. For *Gigaspora* and *Ambispora,* we followed the circumscription of Bentivenga and Morton (1995) and Bills and Morton (2015), respectively. The phenotype of the glomoid spores are indistinguishable among the species of *Ambispora* (Bills and Morton 2015). However, this morphotype can be found in field soils and we decided to add it as an entrance in the glomoid key as *Ambispora* spp. (glomoid morphotype).

Because species descriptions for AMF are not standardized and some fail to clearly indicate layers forming the spore and germinal walls, we faced some problems during the process of obtaining morphological data for species to be included in the taxonomic keys. In these cases, we interpret spore wall and germinal wall phenotypes based on the model of spore development (Morton et al. 1995) and annotation of our interpretation for some species (as they diverge from the original description) are presented in the column “Observations” (Supplementary Material S1). Types of issues encountered during data collection were:

1. Reinterpration of primary characters: terminology used to describe the primary characters of some AMF species was transposed to that of the spore developmental model. For example, Ferrer and Herrera (1980) considered spores being formed by exospore, mesospore and endospore (for example in *Scutellospora trycalipta, Racocetra alborosea, Racocetra minuta*), which were reinterpreted as spore wall, germinal walls 1 and 2, respectively. Oehl et al. (2012) considered spores of *Acaulospora nivalis* (and some other species) formed by outer wall, middle wall, and inner wall, which were reinterpreted as spore wall, germinal 1 and 2, respectively.
2. Inadequate/misinterpretations of wall characters: description of some species fails to clearly show some layers forming the spore wall or germinal wall. For example, we interpreted the germinal wall 2 of *Bulbospora minima* as bilayered as the third layer of this wall is not shown in the illustration and layers 1 and 2 of spore wall of *Dentiscutata colliculosa* was interpreted as only one layer as patterns of spore development in Gigasporaceae indicates only one layer outside the laminated layer. Protologue of *Acaulospora entreriana* indicates the presence of only one germinal wall, despite illustrations in the protologue showing two germinal walls. Revision of the type material also helped to elucidate spore morphology for few species. For both species of *Paradentiscutata*, the protologue indicates the presence of two germinal walls (referred to as middle wall and inner wall), but close inspection of the type material indicates presence of only one germinal wall, with the layer forming the “middle wall” interpreted as part of the spore wall (similar to *Dentiscutata biornata*). *Acaulospora endographis* was described having a complex spore wall with six layers, but close inspection of the two spores of the type material clearly shows the presence of only three layers in the spore wall with the ornamented layer (described as part of the spore wall) readily separated from the spore wall and forming the germinal wall 1.
3. Limits in ability to discriminate some taxa morphologically: one limitation encountered during data collection was the difficulty in discriminating some AMF taxa based solely on spore morphology, as some species displayed overlapping morphological characters. This indicates the possibility of cryptic species (*i.e.*, phylogenetically discrete species that share spore morphology) or the need of synonimization of some species. For example, distinction of *Dentiscutata reticulata* and *Dentiscutata nigerita* was not possible based on spore morphology alone.
4. Taxonomic misplacement in Glomeromycota: the following species were excluded because their protologues clearly indicate that they are not AMF: *Gigaspora lazzarii* Montecchi, Ruini & G. Gross, *Acaulospora terricola* Swarupa, Kunwar & Manohar., and *Entrophospora hexagonii* Rhatwal & Gandhe.

We provide four keys to identify spores at the species level. The first key aims to identify the mode of spore formation. Once decided, readers are directed to specific keys for species identification.

### Key for the mode of spore formation

- 1 a. Spores formed on a bulbous suspensor-like cell (Fig. 2a, 2b) differentiated at the tip of a regularly septated sporogenous hypha_________ Gigasporoid spores (go to key A).
- b. Spores not formed on a bulbous suspensor-like cell _______________________ 2.
- 2 a. Spores with one or two scars, associated with a sporiferous saccule (Fig. 2c, 2 d) whose remnants might or not be present attached to the spore _____ Acaulosporoid spores (go to key B).
- b. Spores formed as an inflation at the tip of a sporogeneous hypha (Fig. 2e, 2f) or at the middle of an undifferentiated hypha and directly attached to the subtending hypha ______________________________________________ Glomoid spores (go to key C).

The following notation was followed in the keys: SW (spore wall), GW (germinal wall(s)), Ln (L = layer, n = number indicating the order of the layer within the SW or GW; *e.g.* L1 indicates the first layer of a SW or GW). Measurements of spore diameter or thickness of a layer are indicated by: (Minimum) Mean (Maximum) (*e.g.* (35) 50 (75) µm).

## Key A for *Gigasporoid* species

**Fig. 3:**
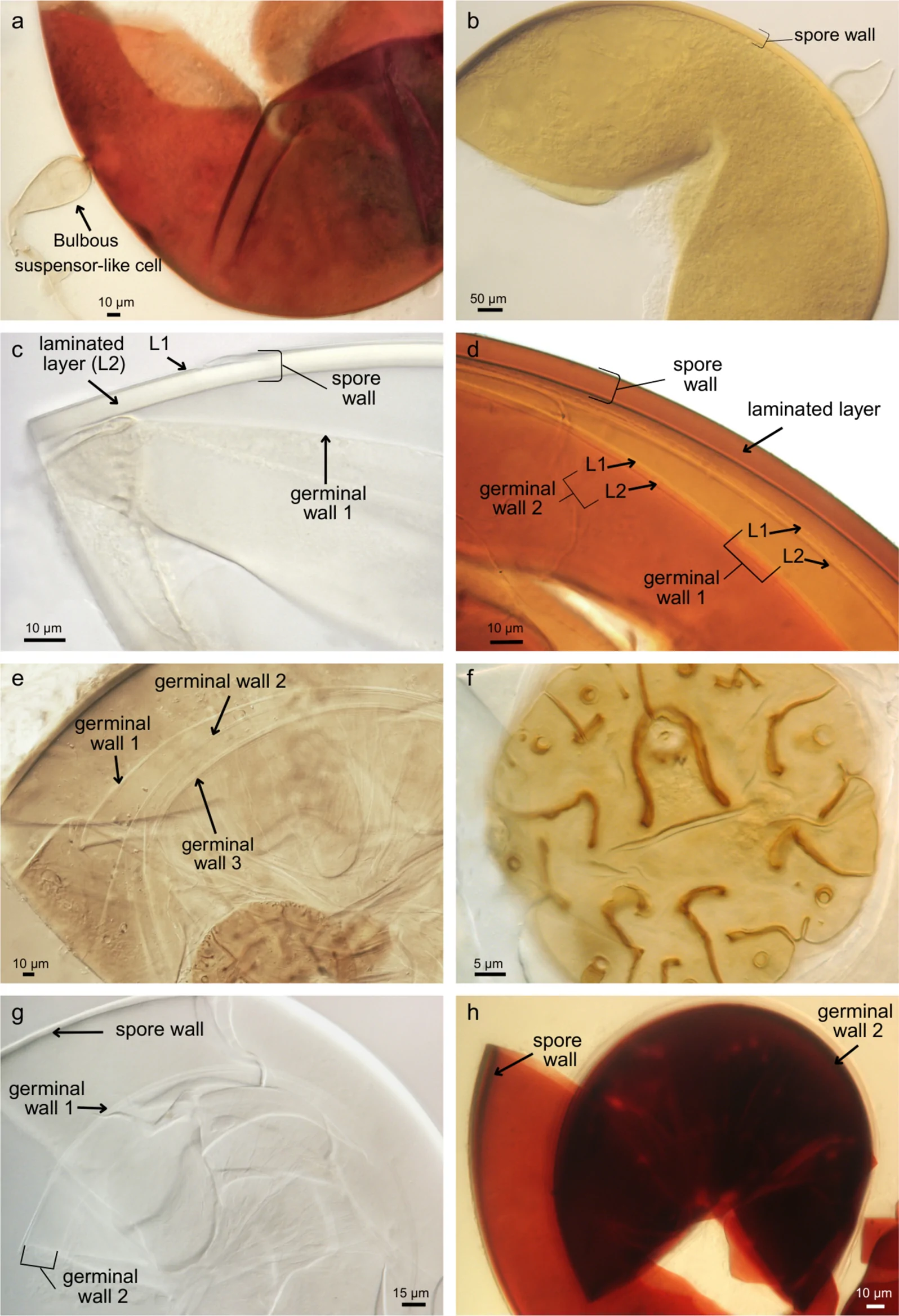
Morphological structures in gigasporoid spores. **a)** bulbous suspensor-like cell of *Gigaspora margarita*. **b)** spore of *Gigaspora albida* formed only by a spore wall. **c)** spore of *Racocetra fulgida* formed by a spore wall (with L1 and laminated layer L2) and one bilayered germinal wall. **d)** spore of *Dentiscutata heterogama* formed by a spore wall showing the laminated layer, and L1 and L2 of both germinal walls (germinal walls 1 and 2).**e)** spore of *Dentiscutata scutata* formed by a spore wall and three germinal walls (germinal walls 1, 2 and 3). **f)** germination shield differentiated by *Dentiscutata scutata*. **g)** spore of *Cetraspora pellucida* mounted in PVLG (notice how germinal walls 1 and 2 are close together). **h)** spore of *Cetraspora pellucida* mounted in PVLG + Melzer’s reagent showing the strong reaction of the spore wall and the second layer of the germinal wall 2

- 1 a. Spores without GW (Fig. 3a, b), not ornamented _______________________________________________________________________ 2

- b. Spores with GW, with or without ornamentation ________________________________________________________________________ 8
- 2 a. Spore diameter ranging from 160—280 µm (mean < 250 µm) _______________________________________________________________ 3
- b. Spore diameter ranging from 240—440 µm (mean > 250 µm) _______________________________________________________________ 4
- 3 a. Spore dull white with a light greenish tint, L1 of SW (2.0) 2.6 (3.2) µm thick, L2 of SW (14.0) 20.0 (26.0) µm thick _____ *Gigaspora albida *(Fig. 3b)
- b. Spore white to cream with a rose-pink tint near the suspensor cell, L1 of SW (1.5) 1.8 (2.2) µm thick, L2 of SW (8.0) 19.0 (29.0) µm thick ___________________________________________________________________________________________________ *Gigaspora rosea*
- 4 a. Spores bright greenish yellow, bright yellow to dark yellow _______________________________________________________________ 5
- b. Spores white to cream, white to orange yellow, yellow brown _____________________________________________________________ 6
- 5 a. Spore bright greenish yellow, color in the spore content, L1 of SW (2.8) 3.2 (3.6) µm thick, L2 of SW (8.0) 17.0 (29.0) µm thick _________ *Gigaspora gigantea*
- b. Spore bright yellow to dark yellow, L1 of SW (1.0) 1.2 (1.5) µm thick, L2 of SW (7.0) 13.0 (28.0) µm thick __________________________ *Gigaspora siqueirae*
- 6 a. Sublayers of L2 not refractive, spores white to cream, L2 of SW (13.0) 22.0 (30.0) µm thick _____________ *Gigaspora margarita* (Fig. 3a)
- b. Sublayers of L2 refracting giving the appearance of a “halo”, spores white to orange yellow or white to cream to yellow brown _________ 7.
- 7 a. Spores white to cream to yellow brown, spores globose to subglobose, L1 of SW (2.5) 2.8 (3.2) µm thick, L2 of SW (15.0) 35.0 (60.0 µm thick ________________________________________________________________________________________ *Gigaspora decipiens*
- b. Spores white to orange yellow, spores irregular, L1 of SW 1.0 µm thick, L2 of SW (10.0) 18.5 (27.0) µm thick ___ *Gigaspora polymorphira*
- 8 a. Spores with 1 GW (Fig. 3c) _______________________________________________________________________________________ 9
- b. Spores with 2 (Fig. 3d) or 3 GW (Fig. 3e) ____________________________________________________________________________ 27
- 9 a. Spore wall smooth ______________________________________________________________________________________________ 10
- b. Spore wall ornamented ___________________________________________________________________________________________ 13
- 10 a. L2 of SW (10.0) 25.0 (35.0) µm thick, spores ochraceous to sienna _________________________________________ *Racocetra castanea*
- b. L2 of SW ranging from 3.0 to 11.0 µm thick (mean < 10.0 µm) __________________________________________________________ 11
- 11 a. SW with 3 layers, spores brown yellow to dark yellow brown, L2 of SW (3.0) 4.8 (6.5) µm thick _________________ *Racocetra tropicana*
- b. SW with 2 layers, spores hyaline to pinkish to pale to slightly darker cream ________________________________________________ 12
- 12 a. Spores hyaline to pinkish, L2 of SW (4.0) 7.5 (11.0) µm thick, spore diameter (204) 245 (287) µm _______________* Racocetra alborosea*
- b. Spores pale to slightly darker cream, L2 of SW (6.0) 7.0 (8.0) µm thick, spore diameter (160) 200 (240) µm ___ *Racocetra fulgida* (Fig. 3c)
- 13 a. Spores with double ornamentation (on the surface AND inside the spore wall) OR only on inside the spore wall ___________________ 14
- b. Spores ornamentation present only on the surface of the spore wall _______________________________________________________ 17
- 14 a. Spores ornamented with tube projections (1.0—2.2 µm long) on the inner surface of L2 of SW, spores bright yellow to yellow orange, L2 of SW (7.5) 10.8 (14.0) µm thick ______________________________________________________________ *Intraornatospora intraornata*
- b. Spores ornamented on the surface AND on inside the spore wall _________________________________________________________ 13
- 15 a. Blunt tapering projections (up to 2 µm high) on the surface of L1 of SW and at the base of L2 of SW, L2 of SW is laminated (5.0) 15.0 (25.0) µm thick, highly plastic, and staining dark red brown to red purple in Melzer ___________________________ *Dentiscutata biornata*
- b. Warts or papillae on the surface of SW and fine tubes or tuberculate-warty projections on the base of SW, L2 of SW always < 5.0 µm thick ___________________________________________________________________________________________ 16
- 16 a. Surface of L1 of SW ornamented with warts (0.8—2.5 µm high) and the base of L2 of SW ornamented with fine tubes (0.5—1.1 µm high), spores dark orange brown to brown _____________________________________________________________ *Paradentiscutata bahiana*
- b. Surface of L1 of SW ornamented with papillae (1.5—2.5 µm high) and the base of L2 of SW ornamented with tuberculate-warty projections (1.0—2.5 µm high) spores bright yellowish brown, orange brown to dark brown _________________ *Paradentiscutata maritima* 

- 17 a. Spores with two types of ornamentations ____________________________________________________________________________ 18
- b. Spores with only one type of ornamentation __________________________________________________________________________ 19
- 18 a. Spores ornamented with warts and blunt bacilliform projections, spores pale dark orange brown, spores (135) 157 (180) µm, L2 of SW (3.0) 5.5 (8.0) µm thick _______________________________________________________________________ *Scutellospora dipapillosa*
- b. Spores ornamented with round warts and outgrowths resembling chromosomes, spores red brown to dark red brown, L2 of SW (9.1) 10.3 (12.4) µm thick ______________________________________________________________________ *Racocetra cromossomica*
- 19 a. Spores ornamented with spines or undulated thickenings ________________________________________________________________ 20
- b. Spores ornamented with warts (Fig. 6g) _____________________________________________________________________________ 21

- 20 a. Spores ornamented with spines, spores dark brown, spore diameter (97) 138 (180) µm, L1 of SW 3.0 µm thick ________ Racocetra minuta
- b. Spores ornamented with undulated thickenings, spores pale yellow, spore diameter (195) 210 (225) µm, L1 of SW (1.4) 1.5 (1.7) µm thick ___________________________________________________________________________ Racocetra undulata
- 21 a. Spores lighter in color: hyaline to white to light ochre __________________________________________________________________ 22
- b. Spores darker in color: dark copper, dark cream, orange red, red brown, orange brown ________________________________________ 23
- 22 a. Spores white to light ochre, L2 of SW (4.5) 7.8 (11.0) µm thick ___________________________________________ *Racocetra beninensis*
- b. Spores hyaline, L2 of SW 5.5 µm thick _________________________________________________________ *Scutellospora trirubiginopa*
- 23 a. Minimum spore diameter 220—240 µm, maximum spore diameter 360 µm and mean spore diameter < 350 µm _____________________ 24
- b. Minimum spore diameter 260—380 µm, maximum spore diameter 480—520 µm and mean spore diameter > 350 µm _________________ 25
- 24 a. Spores pale to dark copper to darker cream, rounded warts 0.5 µm wide, 0.2—0.5 µm high and spaced 0.5—2.0 µm apart __ *Racocetra persica*
- b. Spores pale straw to orange brown, warts 0.5—1.0 µm wide, 0.5—1.2 µm high and spaced 0.5—4.0 µm apart __________ *Racocetra verrucosa*
- 25 a. Spore ornamented with round dome-shaped warts, spores red brown to dark red brown, spore diameter (380) 473 (520) µm_____ *Racocetra gregaria*
- b. Spores ornamented with flattened warts with angular margins or cloud-like warts ___________________________________________ 26
- 26 a. Spores ornamented with flattened warts (Fig. 6g) with angular margins, warts 1—2 µm high, 2—12 µm across, L2 of SW (4.5) 6.2 (8.0) µm thick ______________________________________________________________________________ *Racocetra coralloidea* (Fig. 6g)
- b. Spores ornamented with cloud-like warts, warts 5—18 µm high, 15—41 µm across, L2 of SW (8.0) 10.0 (12.6) µm thick ___ *Racocetra crispa*
- 27 a. Spores with 2 GW (Fig. 3d, g) ___________________________________________________________________________________ 29
- b. Spores with 3 GW (Fig. 3e) ______________________________________________________________________________________ 28
- 28 a. Spores hyaline, spore diameter (340) 494 (640) µm, L2 of SW (3.5) 9.8 (16.0) µm thick _________________ *Dentiscutata scutata* (Fig. 3f)
- b. Spores red-brown to dark red-brown, spore diameter (160) 240 (320) µm, L2 of SW (3.2) 6.8 (7.4) µm thick _____ *Dentiscutata erythropa*
- 29 a. Spore wall smooth ______________________________________________________________________________________________ 30
- b. Spore wall ornamented __________________________________________________________________________________________ 47
- 30 a. Mean spore diameter < 100 µm, spores whitish yellow to light ochre, L2 of SW (2.1) 2.8 (3.5) µm thick ____________ *Bulbospora minima*
- b. Mean spore diameter > 100 µm ___________________________________________________________________________________ 31
- 31 a. Color of spores hyaline, white, or pale to deep pink ____________________________________________________________________ 32
- b. Color of spores in shades of yellow (pale to dark), orange brown (pale to dark), dark brown to black _____________________________ 35
- 32 a. Spores hyaline to white or hyaline to pale and deep pink, with suspensor-like cell hyaline to pale brownish yellow or yellow brown ____ 33
- b. Spores hyaline, with suspensor cell hyaline or brown __________________________________________________________________ 34
- 33 a. Spores hyaline to pale to deep pink, spores globose to subglobose to irregular, spore diameter (125) 210 (414) µm, L2 of SW stains red in Melzer __________________________________________________________________________________ *Scutellospora weresubiae*
- b. Spores hyaline to white, spores predominantly ellipsoid, ovoid or obovoid, spore diameter (175) 467 (760) µm, L2 of SW stains rust red (Fig. 3h) in Melzer ___________________________________________________________________________ *Dentiscutata savannicola*
- 34 a. SW formed by 3 layers, spore diameter (120) 190 (240) µm, suspensor-like cell hyaline _____________ *Cetraspora pellucida* (Fig. 3g, h)
- b. SW formed by 2 layers, spore diameter (204) 262 (320) µm, suspensor-like cell brown _________________________ *Cetraspora gilmorei*
- 35 a. Spores pale yellow with greenish tint _______________________________________________________________________________ 36
- b. Spores in shades of yellow with no greenish tint, pale to dark orange brown, red brown, dark brown to black ______________________ 37
- 36 a. Spore diameter (120) 165 (220) µm, GW1 bilayered _________________________________________________ *Scutellospora calospora*
- b. Spore diameter (140) 176 (240) µm, GW1 monolayered __________________________________________ *Scutellospora dipurpurascens*
- 37 a. Spore color pale to dark orange brown, red brown, dark brown to black ____________________________________________________ 38
- b. Spore color in shades of yellow (pale to dark yellow, golden yellow, brown yellow, ochraceous yellow) __________________________ 40
- 38 a. Spores dark brown to black, spore diameter (303) 345 (387) µm ________________________________________ *Scutellospora tricalypta*
- b. Spore pale to dark orange brown to red brown, spore diameter ranging from 140 to 360 (mean < 300 µm) ________________________ 39
- 39 a. Spore diameter (180) 270 (360) µm, L2 of SW (0.8) 1.5 (2.2) µm thick, L2 of GW2 stains reddish purple in Melzer ___________________ *Dentiscutata hawaiiensis*
- b. Spore diameter (140) 180 (220) µm, L2 of SW (3.5) 7.8 (12.0) µm thick, L2 of GW2 stains pale to dark pinkish purple ________________ *Scutellospora rubra*
- 40 a. Minimum spore diameter 90 to 140 µm, maximum spore diameter 145 to 260 µm and mean spore diameter < 200 µm _______________ 41
- b. Minimum spore diameter 120 to 323 µm, maximum spore diameter 360 to 469 µm and mean spore diameter > 200 µm ______________ 45
- 41 a. L2 of SW ranging from 2.8 to 7.0 µm with mean thickness < 7.0 µm ______________________________________________________ 42
- b. L2 of SW ranging from 5.4 to 13.0 µm with mean thickness > 7.0 µm _____________________________________________________ 43
- 42 a. Spores yellowish gray to light yellow, ovoid or globose, SW formed by 3 layers, L3 of SW stains reddish white to pale red in Melzer _____ *Scutellospora graeca*
- b. Spores yellow ochraceous, ellipsoid, SW formed by 2 layers, layer of spore wall not reacting on Melzer ___________ *Scutellospora ovalis*
- 43 a. Layers of SW not staining in Melzer, spore diameter (110) 127 (145) µm, L2 of SW (7.0) 8.5 (12.0) µm thick ____________ *Scutellospora pernambucana*
- b. L1 or L2 of SW staining in Melzer, spore diameter ranging from 128 to 240 with mean > 150 µm _______________________________ 44
- 44 a. Spores apricot yellow to yellow brown, L1 of SW (0.7) 0.9 (1.2) µm thick, L2 of SW stains garnet red in Melzer _____________________ *Cetraspora armeniaca*
- b. Spores pale yellow to golden yellow, L1 of SW (1.0) 1.5 (2.0) µm thick, L1 of SW stains grayish yellow in Melzer ___________________ *Scutellospora intraundulata*
- 45 a. Spores pale yellow brown to orange brown, spore diameter (120) 240 (360) µm, L2 of SW (3.5) 8.0 (12.0) µm thick, L2 of SW stains dark red brown in Melzer ___________________________________________________________________________ *Scutellospora arenicola*
- b. Spores pale yellow to yellow or bright to golden yellow, spore diameter ranging from 235 to 469 µm (mean > 300 µm) _____________ 46
- 46 a. Spores bright yellow to golden yellow, spore diameter (235) 300 (365) µm, L1 of SW (1.1) 1.4 (1.6) µm thick _____ *Cetraspora auronigra*
- b. Spores pale yellow to yellow, spore diameter (323) 396 (469) µm, L1 of SW (1.0) 3.0 (5.0) µm thick _________ *Scutellospora auriglobosa*
- 47 a. Laminated layer of SW ranging from 1.0 to 5.0 µm thick with mean thickness < 5.0 µm _______________________________________ 48
- b. Laminated layer of SW ranging from 5.0 to 20.0 µm thick with mean thickness > 5.0 µm ______________________________________ 51
- 48 a. SW formed by 1 or 2 layers, spore diameter ranging from 102 to 228 µm with mean spore diameter < 200 µm _____________________ 49
- b. SW formed by 3 layers, spore diameter ranging from 167 to 263 µm with mean spore diameter > 200 µm ________________________ 50
- 49 a. SW bilayered ornamented with spines, spores ochraceous to fulvous or rust, L2 is the laminated layer 2.0 µm thick ___________________ *Cetraspora spinosissima*
- b. SW monolayered ornamented with hooked digitation, rounded bumps or collicles, L1 is the laminated layer (1.0) 1.5 (2.0) µm thick ______ *Scutellospora projecturata*
- 50 a. Spores hyaline to sub-hyaline, L1 of SW ornamented with papillae, L2 of SW (2.0) 3.0 (5.0) µm thick _________ *Dentiscutata cerradensis*
- b. Spores bright yellow to golden yellow, L1 of SW ornamented with tubercles, L2 of SW (1.3) 2.1 (2.9) µm thick ________* Fuscutata aurea*
- 51 a. Spore color in shades of white, hyaline, creamy, ochraceous, or yellow ____________________________________________________ 52
- b. Spore color in shades of dark orange brown, yellow brown (sienna), red to dark brown, or black ________________________________ 56
- 52 a. Spore diameter ranging from 130 to 184 µm and mean spore diameter < 180 µm ____________________________________________ 53
- b. Spore diameter ranging from 150 to 270 µm and mean spore diameter > 180 µm ____________________________________________ 54
- 53 a. Spores ornamented with dome-like subpolygonal papillae, SW formed by 3 layers __________________________ *Scutellospora crenulata*
- b. Spores ornamented with a muri resembling fingerprints, SW formed by 2 layers ______________________________ *Scutellospora striata*
- 54 a. Spores cream white, hyaline to pale yellow, SW ornamented with convex warts or knobby projections, layers of SW not staining in Melzer _____________________________________________________________________________________________________________ 55
- b. Spores yellowish white to bright yellow, SW ornamented with papillae, L2 and L3 of SW stain dark yellow in Melzer and L2 expands to 5.2—8.5 µm in acidic mountants ____________________________________________________________________ *Scutellospora alterata*
- 55 a. Spores creamy white, ornamented with convex warts, SW formed by 3 layers, L2 of SW (5.0) 8.5 (12.0) µm thick and stains dark to black purple in Melzer______________________________________________________________________ *Cetraspora helvetica*
- b. Spores hyaline to pale yellow, ornamented with knobby projections, SW formed by 2 layers, L2 of SW (9.3) 12.4 (16.7) µm thick and stains brownish red in Melzer _______________________________________________________________ *Cetraspora nodosa*
- 56 a. Spore diameter ranging from 120 to 219 µm with mean spore diameter < 250 µm ____________________________________________ 57
- b. Spore diameter ranging from 188 to 1050 µm with mean spore diameter > 250 µm ___________________________________________ 58
- 57 a. SW ornamented with rounded warts, spores dark orange brown to red brown, L2 of SW (5.0) 7.4 (9.0) µm thick and stains dark red brown in Melzer ___________________________________________________________________ *Dentiscutata heterogama* (Fig. 3d)
- b. SW ornamented with annulated pores, spores sienna to fulvous, L2 of SW (7.0) 8.5 (10.0) µm thick not staining in Melzer ____________________________________________________________________________________________ *Scutellospora tepuiensis*
- 58 a. L2 of SW ornamented with papillae and L3 of SW ornamented with undulate colliculate elevations, L2 of SW (10.5) 14.2 (18.0) µm thick _______________________________________________________________________________________ *Dentiscutata colliculosa*
- b. L1 or L2 of SW ornamented with pores, ridges forming reticulum, spines __________________________________________________ 59
- 59 a. Spores dark brown to black, SW pitted with large pores 7—10 µm diameter overlaying smaller pores, L2 of SW (6.0) 13.0 (20.0) µmthick ____________________________________________________________________________________________ *Dentiscutata nigra*
- b. Spores orange brown to dark red brown, SW ornamented with raised ridges forming a reticulum and covered with spines (Fig. 6a), rods, hyphal coils or tree-like structures ___________________________________ *Dentiscutata nigerita/Dentiscutata reticulata* (Fig. 6a)

## Key B for *Acaulosporoid* species

**Fig. 4:**
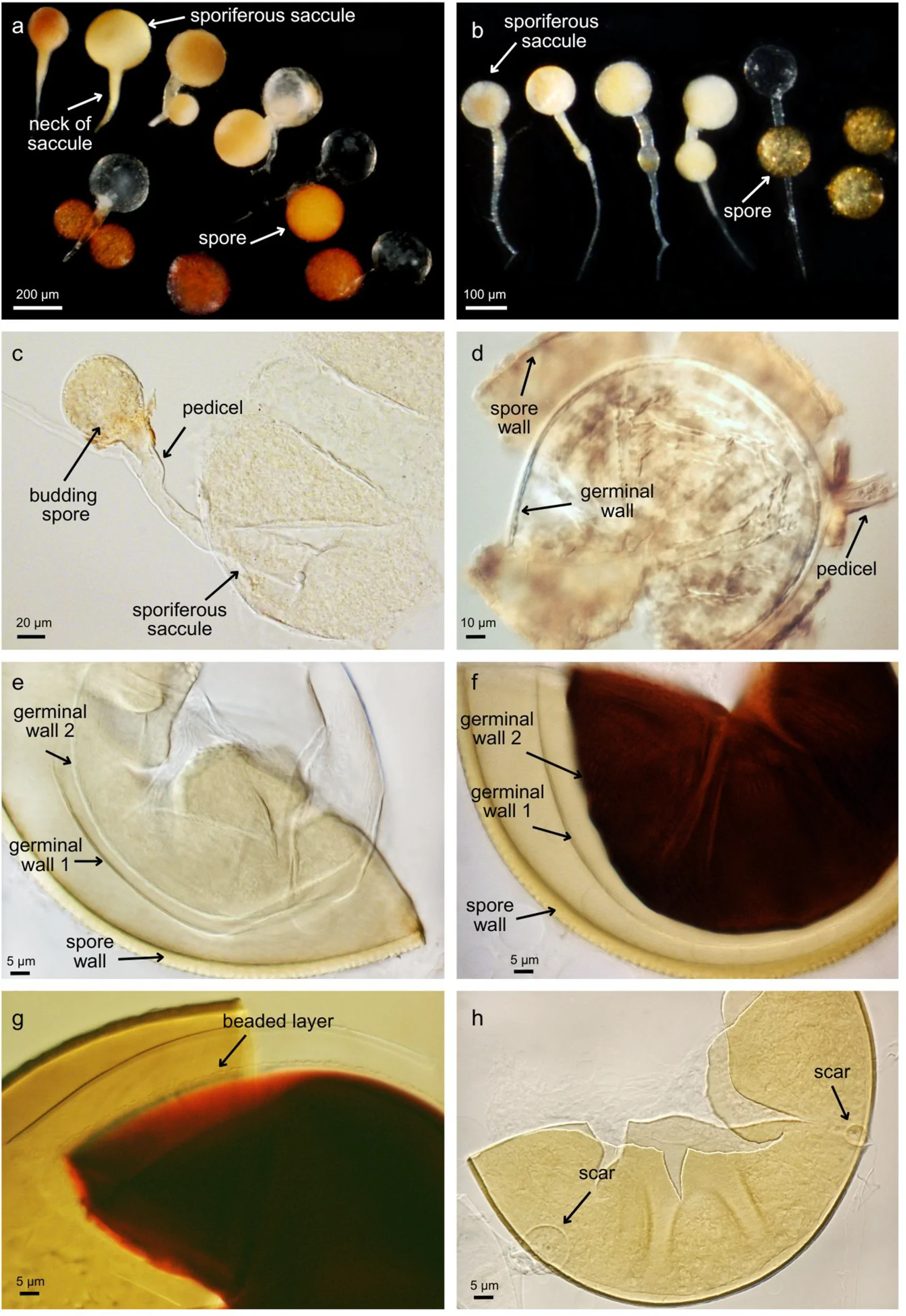
Morphological structures in glomoid spores. **a)** spore of *Glomus macrocarpum* differentiating only a spore wall with two layers (L1 and laminated L2); notice the blastically-formed spore on the sporogenous hypha. **b)** spore of *Pacispora* sp. differentiating a spore wall and a germinal wall. **c)** spore of *Rhizophagus intraradices* differentiating three layers (L1, L2, and L3) in the spore wall and mounted in PVLG. **d)** spore of *Rhizophagus intraradices* mounted in PVLG + Melzer’s reagent showing the reaction of L1. **e)** spore of *Paraglomus occultum* mounted on PVLG + Melzer’s reagent showing no reaction. **f)** spore of *Halonatospora pansihalos* showing the outer most layer expanding in acidic mountant. **g)** spore of *Sieverdingia tortuosa* showing the hyphal mantle covering the spore. **h)** spore of *Funneliformis mosseae* showing sloughing layers (strongly reacting in Melzer’s reagent) detaching from the rigid laminated layer

**Fig. 5:**
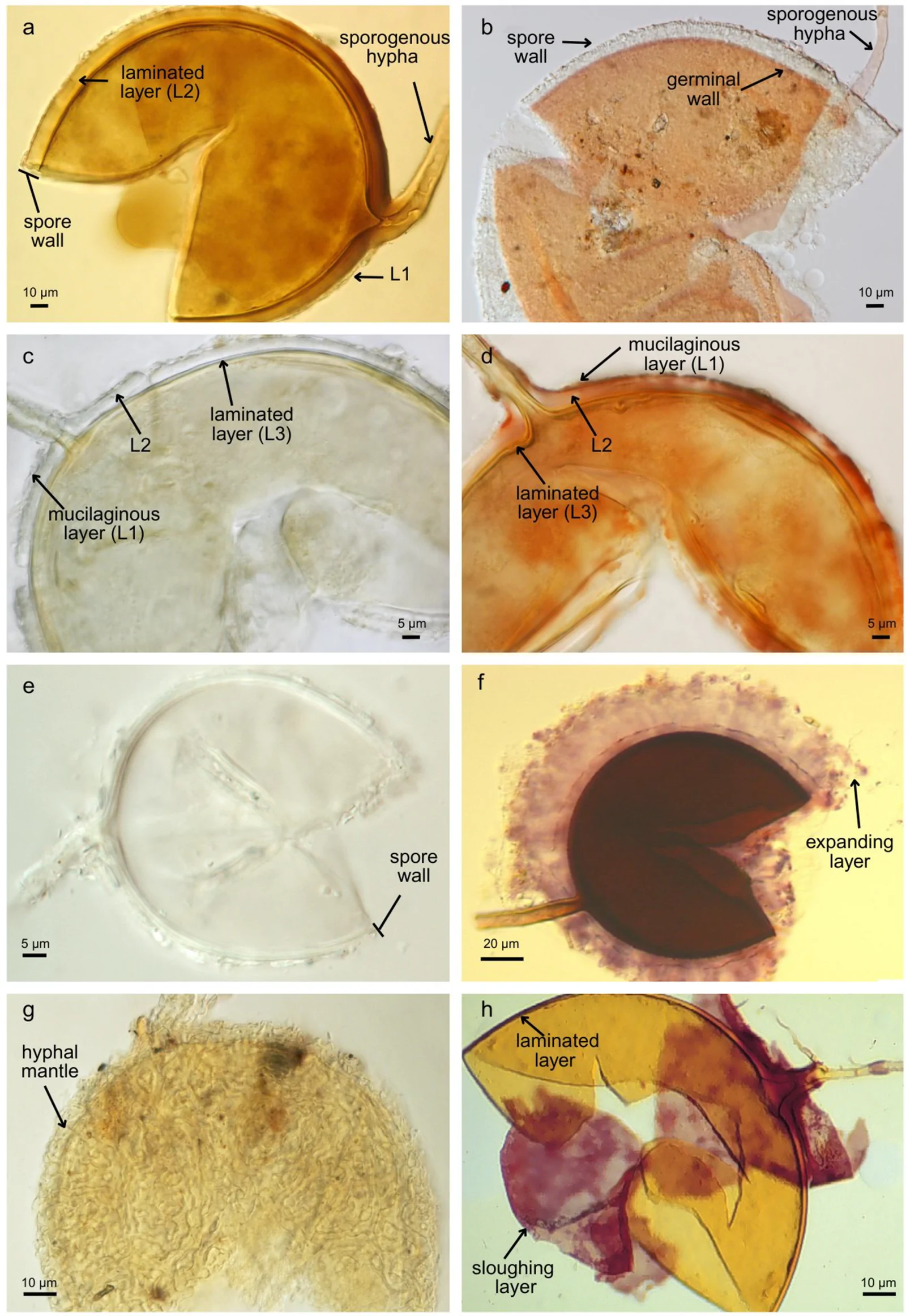
Morphological structures in acaulosporoid spores. **a)** stages of spore development in *Acaulospora foveata* showing the sporiferous saccule and the formation of the spore on the side of the saccule’s neck. **b)** stages of spore development in *Acaulospora colombiana* showing the sporiferous saccule and the formation of the spore within the saccule’s neck. **c)** budding spore of *Ambispora leptoticha* formed at the tip of a pedicel branching from the saccule’s neck. **d)** spore of *Ambispora leptoticha* formed by a spore wall and one germinal wall. **e)** spore of *Acaulospora* sp. formed by a spore wall and two germinal walls (germinal walls 1 and 2) mounted in PVLG. **f)** spore of *Acaulospora* sp. formed by a spore wall and two germinal walls (germinal walls 1 and 2) mounted in PVLG + Melzer’s reagent showing the strong reaction of the second layer of the germinal wall 2. **g)** spore of *Acaulospora lacunosa* showing the beaded layer of germinal wall 2. **h)** spore of *Acaulospora colombiana* showing two scars
- 2 a. Spore diameter ranging from 22 to 99 µm and mean spore diameter < 100 µm, hyaline and subhyaline to grayish white to light and orange yellow, SW always formed by 2 layers ________________________________________________________________________________ 3
- b. Spore diameter ranging from 87 to 260 µm and mean spore diameter > 100 µm, mostly in shades of yellow, orange, brown or black, less often hyaline to white to cream, SW formed by 2, 3 or 4 layers ____________________________________________________________ 11
- 3 a. SW ornamented __________________________________________________________________________________________________ 4
- b. SW smooth (not ornamented) _______________________________________________________________________________________ 5
- 4 a. Spores light yellow to orange yellow, ornamented with ridges forming a reticulum, L2 of SW (4.0) 5.0 (6.0) µm thick __________________ *Acaulospora taiwania*
- b. Spores hyaline to subhyaline, ornamented with pits, L2 of SW (1.0) 1.3 (1.5) µm thick ________________________ *Archaeospora undulata*
- 5 a. GW formed by 2 layers ____________________________________________________________________________________________ 6
- b. GW formed by 1 or 3 layers ________________________________________________________________________________________ 7
- 6 a. GW (1.9) 2.7 (3.4) µm thick, L2 of SW 0.2—2.2 µm with fissure and staining yellowish to pink yellowish in Melzer ____________________ *Archaeospora europaea*
- b. GW (1.4) 1.5 (2.0) µm thick, L2 of SW 0.8—1.0 µm without fissures and not staining in Melzer _____________________________________ *Antiquispora disseminans*
- 7 a. Spores with two cicatrices (scars), hyaline, GW (1.3) 2.7 (4.0) µm thick ___________________________________ *Archaeospora schenckii*
- b. Spores with one cicatrix (scar) ______________________________________________________________________________________ 8
- 8 a. Spores formed in sporocarps or singly in the soil, spore diameter (22) 32 (90) µm, L1 of SW (0.7) 1.4 (2.0) µm thick ___________________ *Archaeospora myriocarpa*
- b. Spores formed only singly in the soil, spore diameter ranging from 40 to 99 µm and mean spore diameter > 50 µm ___________________ 9
- 9 a. SW formed by 3 layers, L2 of SW 0.9—3.2 µm thick with fissures, L2 stains bright yellow in Melzer _____________ *Archaeospora spainiae*
- b. SW formed by 2 layers ___________________________________________________________________________________________ 10
- 10 a. L2 of SW 3.0 µm thick, spores stain yellow in Melzer but reaction fades soon ____________________________ *Andinospora ecuadoriana*
- b. L2 of SW < 1.0 µm thick, spores not staining in Melzer _________________________________________________ *Archaeospora trappei*
- 11 a. SW with 2 layers _______________________________________________________________________________________________ 12
- b. SW with 3 or 4 layers ___________________________________________________________________________________________ 14
- 12 a. Spores hyaline, spore diameter (190) 220 (250) µm, GW formed by 3 layers, L3 of GW stains reddish brown in Melzer ________________ *Acaulospora splendida*
- b. Spores with shades of brown to black, mean spore diameter 170 to 180 µm, GW formed by 1 or 2 layers _________________________ 13
- 13 a. Spores dark brown to black, L2 of SW (2.5) 3.8 (5.0) µm thick ________________________________________ *Acaulospora sporocarpia*
- b. Spores pale to yellow–brown to brown, L2 of SW (1.0) 1.5 (2.0) µm thick ___________________________________ *Acaulospora walkeri*
- 14 a. Spores with two cicatrices (or scars) ________________________________________________________________________________ 15
- b. Spores with one cicatrix (or scar) __________________________________________________________________________________ 18
- 15 a. Spores not enveloped by hyphal mantle _____________________________________________________________________________ 16
- b. Spores enveloped by hyphal mantle ________________________________________________________________________________ 17
- 16 a. Spores ornamented with tooth-shaped outgrowths, L1 of SW (2.0) 3.5 (5.0) µm thick, L1 of SW stains pink to dark pink in Melzer _________________________________________________________________________________________________ *Entrophospora infrequens*
- b. Spores ornamented with spiny to thorn-like curved projections, L1 of SW (0.6) 1.2 (1.8) µm thick, L1 of SW stains pinkish in Melzer ____ ___________________________________________________________________________________________ *Diversispora nevadensis*
- 17 a. L2 of SW ornamented with warts 0.6—0.8 µm high, L3 of SW laminated (1.5) 2.1 (2.7) µm thick _________________ *Sacculospora baltica*
- b. L2 of SW ornamented with warts 1.0—4.0 µm high, L3 of SW rigid (1.5) 2.6 (4.0) µm thick ____________________ *Sacculospora felinovii*
- 18 a. SW smooth (not ornamented) (Fig. 5g, h) ___________________________________________________________________________ 19
- b. SW ornamented (Fig. 5e, f) ______________________________________________________________________________________ 21
- 19 a. SW with 4 layers, L3 of SW (2.5) 4.0 (6.0) µm thick ____________________________________________________ *Diversispora bareae*
- b. SW with 3 layers, L3 of SW ranging from 0.5 to 2.5 µm (mean < 4.0 µm) __________________________________________________ 20
- 20 a. Spore diameter (160) 213 (260) µm, L1 of SW (6.0) 10.5 (15.0) µm thick, L3 of SW brittle and breaking into shards birefringent in polarized light ________________________________________________________________________________ *Ambispora gerdemannii*
- b. Spore diameter (99) 158 (218) µm, L1 of SW (0.5) 0.8 (1.0) µm thick, L3 of SW not brittle _____________________ *Ambispora nicolsonii*
- 21 a. Spores cream to dark orange brown, spore diameter (160) 205 (240) µm, L2 and L3 of SW forming hemispherical convex and concave protuberances (Fig. 5h), respectively ___________________________________________________ *Ambispora leptoticha* (Fig. 4c, d, 6h)

- 1 a. Spores with 1 GW (Fig. 4d) ________________________________________________________________________________________ 2

- b. Spores with 2 GW (Fig. 5e, f) _____________________________________________________________________________________ 23

**Fig. 6:**
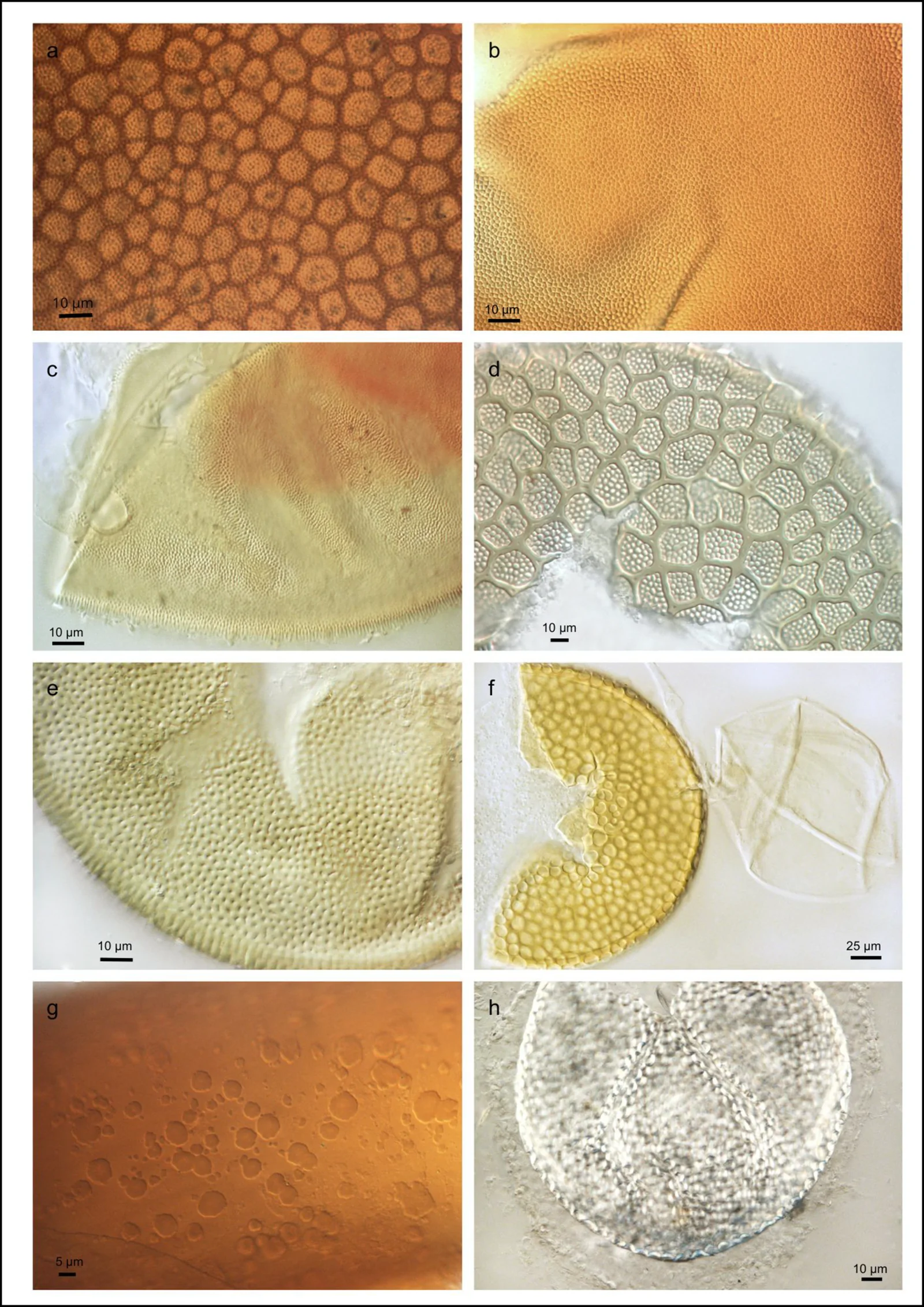
Examples of ornamentation patterns found in AMF spores. **a)** raised ridges forming a reticulum and covered with spines of *Dentiscutata reticulata*. **b)** tubercles of *Acaulospora tuberculata*. **c)** spines of *Acaulospora spinosa*. **d)** alveolate reticulum superimposed on spines of *Acaulospora elegans*. **e)** pits of *Acaulospora scrobiculata*. **f)** large pits of *Acaulospora* sp. **g)** flattened warts of *Racocetra coralloidea*. **h)** hemispherical convex and concave protuberances of *Ambispora leptoticha*
- b. Spores hyaline to white to white yellow or yellow brown to brown, mean spore diameter < 150 µm, SW ornamented __________ 22
- 22 a. Spores hyaline to white to white yellow, L1 of SW ornamented with papillae-like protrusions, L2 of SW (0.8) 1.2 (1.5) µm thick _____________________________________________________________________________________________________ *Ambispora granatensis*
- b. Spores yellow brown to brown, L2 of SW ornamented with a reticulate ornamentation, L2 of SW (3.0) 5.5 (8.0) µm thick ______________ ______________________________________________________________________________________________ *Ambispora reticulata*
- 23 a. Spores with two cicatrices (or scars) (Fig. 5h) ________________________________________________________________________ 24
- b. Spores with one cicatrix (or scar) __________________________________________________________________________________ 28
- 24 a. SW smooth (not ornamented) _____________________________________________________________________________________ 25
- b. SW ornamented with pits, spines or protrusions _______________________________________________________________________ 26
- 25 a. Spore diameter (100) 121 (140) µm, spore globose to subglobose, L2 of SW (2.4) 3.3 (4.4) µm thick_ *Acaulospora colombiana* (Fig. 5b,h)
- b. Spore diameter (64) 81 (99) µm, spores subglobose to ellipsoid, L2 of SW (3.3) 4.3 (5.4) µm thick ________________ *Acaulospora tsugae*
- 26 a. Spores ornamented with pits, spores pale yellow to yellow brown, L2 of SW (2.9) 3.4 (4.4) µm thick ___________ *Acaulospora kentinensis*
- b. Spores ornamented with spines or protrusions, hyaline to subhyaline to pale yellow and yellowish white __________________________ 27
- 27 a. Spore diameter (75) 84 (100) µm, spores ornamented with protrusions, layers of SW and GW not staining in Melzer ______________________________________________________________________________________________________________ *Acaulospora colliculosa*
- b. Spore diameter (100) 175 (250) µm, spores ornamented with spines, L2 of GW2 stains pink to reddish purple in Melzer ______________________________________________________________________________________________________________ *Kuklospora spinosa*
- 28 a. Spores with GW2 not reacting on Melzer ____________________________________________________________________________ 29
- b. Spores with GW2 staining pinkish to red dark purple to red brown or light greenish yellow ochre in Melzer _______________________ 34
- 29 a. SW not ornamented (smooth) _____________________________________________________________________________________ 30
- b. SW ornamented _______________________________________________________________________________________________ 31
- 30 a. Spores brown, spore diameter (280) 290 (300) µm, L2 of SW (1.2) 2.7 (4.6) µm thick _______________________ *Acaulospora entreriana*
- b. Spores pale yellow to lemon yellow, spore diameter (55) 65 (75) µm, L2 of SW (2.2) 3.6 (4.3) µm thick ________ *Acaulospora gedanensis*
- 31 a. SW ornamented with pustules _____________________________________________________________________________________ 32
- b. SW ornamented with fibrillose hairy outgrowth or hypha-like structures ___________________________________________________ 33
- 32 a. Spores hyaline to subhyaline, ornamented with convex pustules, L2 of SW (2.2) 2.7 (3.2) µm thick ____________* Acaulospora brasiliensis*
- b. Spores yellow brown, ornamented with pustulate projections, L2 of SW (1.5) 1.9 (2.3) µm thick ________________ *Acaulospora pustulata*
- 33 a. Spores pale brown to brown, L2 of SW (1.2) 1.4 (1.6) µm thick ornamented with fibrillose hairy outgrowth________ *Acaulospora soloidea*
- b. Spores orange brown, L1 of SW (2.0) 2.6 (3.2) µm thick ornamented with hyphae-like structures ________________ *Acaulospora tortuosa*
- 34 a. SW not ornamented (smooth) ____________________________________________________________________________________ 65
- b. SW ornamented _______________________________________________________________________________________________ 35
- 35 a. SW ornamented with pits (Fig. 6e, f) or spines (Fig. 6c) _______________________________________________________________ 36
- b. SW ornamented with other types of ornamentation (depressions, projections, reticulum, papillae) (Fig. 6a, b, d) __________________ 57
- 36 a. Spores ornamented with pits ______________________________________________________________________________________ 37
- b. Spores ornamented with spines ____________________________________________________________________________________ 53
- 37 a. Spore diameter generally 50—97 µm, rarely up to 112 µm, with mean spore diameter always < 100 µm ___________________________ 38
- b. Spore diameter generally 103—360 µm, rarely as small as 80 µm, with mean spore diameter always > 100 µm _____________________ 44
- 38 a. Spores hyaline to subhyaline to white to pale yellow to cream ___________________________________________________________ 39
- b. Spores yellow (bright to dark) to orange to brown to creamy to light brown ________________________________________________ 41
- 39 a. Spores pale yellow to cream, pits round, elliptical to oblong, with pit size from 3.2—16.2 µm in diameter _____________ *Acaulospora verna*
- b. Spores hyaline to subhyaline to white to pale yellow, pits round to elliptical, with pit size from 0.5—3.5 µm in diameter ______________ 40
- 40 a. L2 of SW (1.5) 2.0 (2.5) µm thick, L2 of GW2 stains pale red in Melzer, pits 2.0—3.0 × 2.5—3.5 µm diameter ______ *Acaulospora paulineae*
- b. L2 of SW (3.0) 4.5 (6.0) µm thick, L2 of GW2 stains purple to dark purple in Melzer, pits 0.8—1.8 µm diameter when round 0.5—1.2 × 1.5—2.5 µm diameter when oblong ________________________________________________________ *Acaulospora sieverdingii*
- 41 a. Pits with regular round shape _____________________________________________________________________________________ 42
- b. Pits with round to elliptical, vermiform, or oblong shape ________________________________________________________________ 43
- 42 a. Spores dark yellow, orange to brown, pits (1.5) 2.2 (2.8) µm in diameter, L2 of SW (2.0) 2.5 (3.0) µm thick ________ *Acaulospora alpina*
- b. Spores cream to light brown, with grayish tint, pits (0.7) 1.2 (1.6) µm in diameter, L2 of SW (2.7) 3.8 (4.0) µm thick _ *Acaulospora baetica*
- 43 a. Spores yellowish brown, pits round (0.5—1.4 µm diameter) to elliptical (1.3—1.9 µm long and 0.9—1.4 µm wide) to vermiform (2.2—4.8 µm long and 0.5—1.0 µm wide) ______________________________________________________________ *Acaulospora herrerae*
- b. Spore bright to dark yellow, pits regular to highly irregular, round to slightly edged, oblong 0.9—1.5 x (0.9-) 1.5—3.5 (−5.5) µm in diameter ______________________________________________________________________________________ *Acaulospora nivalis*
- 44 a. Spores red to dark orange to dark red–orange brown ___________________________________________________________________ 45
- b. Spores yellow to light brown, ochraceous, green or turquoise, yellow white to cream, subhyaline to pale yellow to straw colored ______ 47
- 45 a. Spore diameter > 240 µm (240—360 µm), L2 of SW (9.0) 13.0 (18.0) µm thick ________________________ *Acaulospora foveata* (Fig. 5a)
- b. Spore diameter < 240 µm, L2 of SW 1.6—6.0 µm thick _________________________________________________________________ 46
- 46 a. Spores reddish yellow, spore diameter (80) 120 (130) µm, pits saucer-shaped or cone-shaped 0.2—3.0 × 0.2—6.0 µm in diameter ______________________________________________________________________________ *Acaulospora lacunosa* (Fig. 5g)
- b. Spores dark orange to dark orange brown, spore diameter (150) 175 (230) µm, pits regular or slightly irregular (0.5) 1.2 (1.8) µm in diameter ______________________________________________________________________ *Acaulospora minuta*
- 47 a. Pits mostly of regular circular to subcircular shape ____________________________________________________________________ 48
- b. Pits of irregular shape ___________________________________________________________________________________________ 52
- 48 a. Laminated layer < 4.5 µm thick ___________________________________________________________________________________ 49
- b. Laminated layer > 4.5 µm thick ___________________________________________________________________________________ 50
- 49 a. Spores yolk yellow to light brown, L2 of SW (1.2) 2.0 (3.4) µm thick with round pits 2.0—5.0 µm in diameter _____ *Acaulospora cavernata*
- b. Spores yellow white to creamy, L2 of SW (2.0) 3.2 (4.3) µm thick with round pits (1.1) 2.0 (2.7) µm in diameter ___ *Acaulospora punctata*
- 50 a. L2 of SW (4.5) 6.1 (7.0) µm thick, spores light olive to light brown, widest pits diameter 1.0—3.0 µm __ *Acaulospora scrobiculata* (Fig. 6e)
- b. L2 of SW ranging from 8.0 to 11.0 µm thick (mean > 7.0 µm) ___________________________________________________________ 51
- 51 a. Spores pale yellow to pale yellowish brown to straw, L2 of SW (8.0) 8.5 (9.0) µm thick with pits 2.2—7.7 µm diameter ___________________________________________________________________________________________________________ *Acaulospora guangxiensis*
- b. Spores pale ochraceous to ochre to orange, L2 of SW (8.0) 9.5 (11.0) µm with pits 4.0—20.0 µm diameter _________ *Acaulospora excavata*
- 52 a. Spores green or turquoise, pits polygonal or irregularly ellipsoid 1.3—2.4 µm in diameter, L2 of SW (4.1) 5.5 (6.8) µm thick __________________________________________________________________________________________________________ *Acaulospora jejuensis*
- b. Spores whitish to dark yellow, pits edged to dumbbell shaped 5.5—19.0 × 3.5—8.6 µm in diameter, L2 of SW (6.0) 10.3 (14.5) µm thick __________________________________________________________________________________________________ *Acaulospora reducta*
- 53 a. Spores ornamentation (spines) present in the L1 of GW1 _____________________________________________ *Acaulospora endographis*
- b. Spores ornamentation (spines) present in the L2 of SW _________________________________________________________________ 54
- 54 a. Spore diameter 60—120 µm and mean spore diameter < 120 µm __________________________________________________________ 55
- b. Spore diameter 116—220 µm and mean spore diameter > 120 µm _________________________________________________________ 56
- 55 a. SW with 2 layers, L2 of SW (3.0) 3.5 (4.0) µm thick _________________________________________________ *Acaulospora mendoncae*
- b. SW with 3 layers, L2 of SW (1.0) 1.9 (2.8) µm thick _________________________________________________ *Acaulospora spinulifera*
- 56 a. Spores bright yellow to brownish yellow, L2 of SW (1.0) 1.8 (2.7) µm thick, spines 0.5—1.1 µm high _________ *Acaulospora spinosissima*
- b. Spores yellowish brown to dark reddish brown, L2 of SW (6.0) 7.5 (8.5) µm thick including spines, spines 1.0—4.0 µm high ____________________________________________________________________________________________________ *Acaulospora spinosa* (Fig. 6c)
- 57 a. Spores ornamented with papillae or depressions _______________________________________________________________________ 58
- b. Spores ornamented with projections or reticulum ______________________________________________________________________ 61
- 58 a. Spores ornamented with papillae in the L1 of the SW __________________________________________________________________ 59
- b. Spores ornamented with depressions in the L2 of SW __________________________________________________________________ 60
- 59 a. Spores yellow white to light yellow to creamy, spore diameter (65) 97 (110) µm, L2 of SW (1.7) 3.0 (4.3) µm thick ________________________________________________________________________________________________________________ *Acaulospora papillosa*
- b. Spores brownish yellow to yellow brown, spore diameter (125) 142 (160) µm, L2 of SW (3.0) 4.0 (5.0) µm thick ______________________________________________________________________________________________________________ *Acaulospora flavopapillosa*
- 60 a. Spores ornamented with depressions 0.4—0.7 µm in diameter, 0.8 µm deep, with washboard-like appearance, L1 of SW (1.6) 2.3 (3.1) µm thick, L2 of SW (2.6) 3.7 (4.9) µm thick ______________________________________________________________ *Acaulospora aspera*
- b. Spores ornamented with depressions 0.5—0.8 µm in diameter, 0.5—1.4 µm deep, creating a network of ridges in a complex labyrinthian pattern, L1 of SW (0.8) 1.0 (1.2) µm thick, L2 of SW (4.5) 10.0 (12.0) µm thick _______________________________ *Acaulospora rehmii*
- 61 a. Spores ornamented with a reticulum ________________________________________________________________________________ 62
- b. Spores ornamented with projections ________________________________________________________________________________ 63
- 62 a. Spores light brown, spore diameter (150) 153 (155) µm, ornamented with polygonal reticulum enclosing round-tipped processes, polygons 6—18 µm long separated by ridges 1.5—2.0 × 2.0 µm __________________________________________________ *Acaulospora bireticulata*
- b. Spores dark brown, spore diameter (140) 235 (330) µm, ornamented with alveolate reticulum superimposed on spines, alveoli 4—8 µm long separated by ridges 1.0 × 5.0—6.0 µm _________________________________________________________ *Acaulospora elegans* (Fig. 6d)
- 63 a. Spores ornamented with warts or flattened elevations, SW formed by 2 layers, spore diameter (65) 72 (80) µm _______ *Acaulospora ignota*
- b. Spores ornamented with circular to oblong projections or tubercles, SW formed by 3 layers, spore diameter > 100 µm ______________ 64
- 64 a. Spores yellow brown to dark brown, spores ornamented with circular to oblong projections with a center cavity (denticulate ornamentation), L2 of SW (1.0) 1.2 (1.4) µm thick without the ornamentations ____________________________ *Acaulospora denticulata*
- b. Spores dark honey brown to reddish black, spores ornamented with tubercles (Fig. 6b), L2 of SW (8.5) 9.2 (11.8) µm thick ________________________________________________________________________________________________ *Acaulospora tuberculata* (Fig. 6b)
- 65 a. Minimum spore diameter 55 to 109 µm and maximum spore diameter 83 to 160 µm, mean spore diameter < 150 µm ________________ 66
- b. Minimum spore diameter 120 to 180 µm and maximum spore diameter 205 to 380 µm and mean spore diameter > 150 µm ___________ 79
- 66 a. Thickness of laminated layer ranging from 1.7—3.9 µm, with mean thickness ≤ 3.0 µm ________________________________________ 67
- b. Thickness of laminated layer ranging from 2.0—6.5 µm, with mean thickness > 3.0 µm ________________________________________ 73
- 67 a. Spores reddish orange to reddish brown, spore diameter (109) 123 (138) µm, L2 of SW (2.0) 2.7 (3.5) µm thick _____ *Acaulospora fanjing*
- b. Spores light in color: hyaline, subhyaline, light yellow, pale yellowish cream, pale ochraceous to pale yellow brown ________________ 68
- 68 a. SW with 2 layers _______________________________________________________________________________________________ 69
- b. SW with 3 layers _______________________________________________________________________________________________ 71
- 69 a. Spores creamy white to pale ochraceous, spore diameter (60) 67 (83) µm, L1 of GW2 stains light greenish yellow ochre tint in Melzer _____________________________________________________________________________________ *Acaulospora fragilissima*
- b. Spores hyaline/subhyaline to pale yellow, spore diameter ranging from 55 to 150 µm (mean > 90 µm), L2 of GW stains pink to purple in Melzer _____________________________________________________________________________________________ 70
- 70 a. Spore diameter (80) 115 (150) µm, L1 of SW (0.4) 0.6 (0.8) µm thick, L2 of GW2 stains light to darker pink in Melzer, occasionally showing no reaction _____________________________________________________________________________ *Acaulospora delicata*
- b. Spore diameter (55) 83 (100) µm, L1 of SW (0.5) 1.7 (3.0) µm thick, L2 of GW2 stains light purple in Melzer ______ *Acaulospora longula*
- 71 a. Spores light yellow, L1 of SW (1.0) 1.2 (1.5) µm thick _______________________________________________ *Acaulospora jiangxiensis*
- b. Spores subhyaline to pale yellowish brown, L1 of SW ranging from 0.5 to 1.2 µm thick (mean < 1.0 µm) _________________________ 72
- 72 a. L2 of SW (2.0) 2.2 (2.4) µm thick ________________________________________________________________ *Acaulospora morrowiae*
- b. L2 of SW (2.0) 2.4 (3.2) µm thick ___________________________________________________________________ *Acaulospora rugosa*
- 73 a. SW with 2 layers _______________________________________________________________________________________________ 74
- b. SW with 3 layers _______________________________________________________________________________________________ 75
- 74 a. Spores white to pale ochraceous, spore diameter (69) 75 (85) µm, L2 of SW (4.0) 5.5 (6.5) µm thick, L2 of GW2 stains deep beetroot purple in Melzer _________________________________________________________________________________ *Acaulospora saccata*
- b. Spores hyaline to white, spore diameter (80) 94 (115) µm, L2 of SW (3.3) 4.7 (5.5) µm thick, L2 of SW stains reddish white in Melzer _____________________________________________________________________________________ *Polonospora polonica*
- 75 a. Spores pale to deep yellow, bright yellow to yellow brown, mean spore diameter < 120 µm ____________________________________ 76
- b. Spores light to dark brown, honey colored to yellow brown, mean spore diameter ≥ 120 µm ___________________________________ 78
- 76 a. Spores pale yellow, spore diameter (70) 85 (100) µm __________________________________________________ *Acaulospora citrusnsis*
- b. Spores deep to bright yellow to yellow brown, spore diameter ranging from 95 to 160 µm (mean > 100 µm) ______________________ 77
- 77 a. Spores deep yellow, spore diameter (100) 113 (130) µm, L2 of SW (2.8) 4.7 (5.5) µm thick _____________________ *Acaulospora dilatata*
- b. Spores bright yellow to yellow brown, spore diameter (95) 105 (160) µm, L2 of SW (2.6) 3.7 (4.9) µm thick _________ *Acaulospora flava*
- 78 a. Spores light to dark brown, spore diameter (99) 132 (158) µm, L2 of GW2 stains light pink in Melzer ____________ *Acaulospora koreana*
- b. Spores honey colored to yellow brown, spore diameter (90) 120 (140) µm, L2 of GW2 stains red purple to dark red purple in Melzer ____________________________________________________________________________________________________ *Acaulospora mellea*
- 79 a. L3 of SW staining in Melzer ______________________________________________________________________________________ 80
- b. L3 of SW not staining in Melzer ___________________________________________________________________________________ 81
- 80 a. Spores pale yellow to yellow, L3 of SW stains pale reddish purple in Melzer ___________________________ *Acaulospora intravesiculata*
- b. Spores pale yellow brown to dark orange brown, L3 of SW stains orange brown to dark red brown in Melzer ________ *Acaulospora koskei*
- 81 a. Spore diameter ranging from 170 to 380 µm and mean spore diameter > 250 µm ____________________________________________ 82
- b. Spore diameter ranging from 140 to 240 µm and mean spore diameter > 150 and < 250 µm ____________________________________ 83
- 82 a. Spores orange red to capsicum red, L2 of SW (2.2) 4.2 (5.6) µm thick ____________________________________ *Acaulospora capsicula*
- b. Spores orange brown to dark red brown, L2 of SW (2.0) 3.2 (4.8) µm thick ________________________________ *Acaulospora colossica*
- 83 a. L2 of SW (4.9) 6.7 (8.5) µm thick, L1 of GW1 stains light purple to purple in Melzer, GW2 formed by 3 layers ______ *Acaulospora viridis*
- b. L2 of SW ranging from 1.6 to 4.0 (mean < 4.0 µm), L1 of GW1 not staining in Melzer, GW2 formed by 2 layers ___________________ 84
- 84 a. Spores salmon to orange brown, most pale orange brown, L1 of SW (1.2) 1.6 (2.0) µm thick, L2 of SW (1.6) 1.9 (2.8) µm thick _________________________________________________________________________________________________________ *Acaulospora laevis*
- b. Spore dark brown to black, L1 semipermanent (2.5) 4.4 (6.5) µm thick and L2 (2.5) 3.3 (4.0) µm thick _____________ *Acaulospora thomii*

## Key C for *Glomoid spores*

**Fig. 7:**
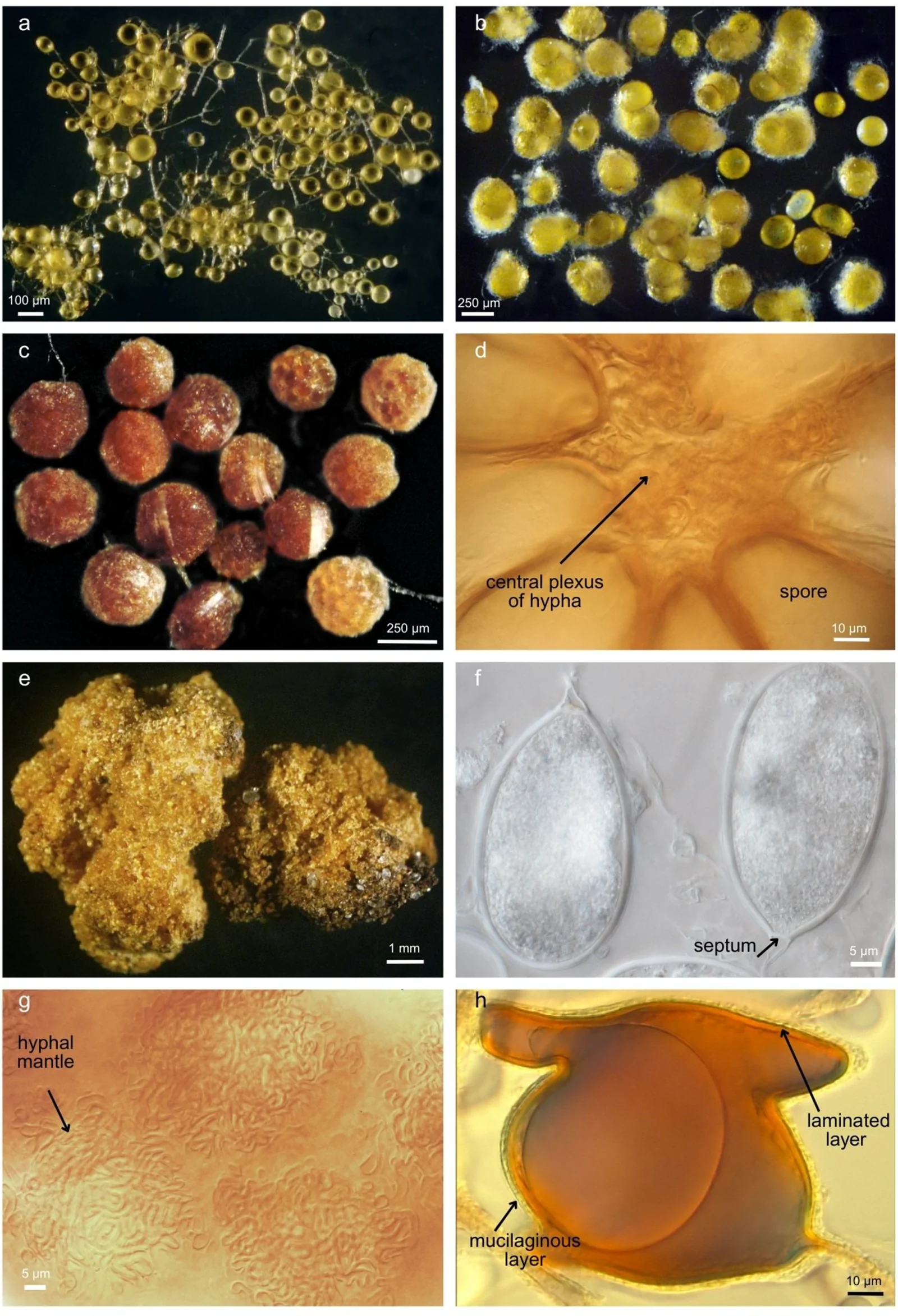
**a)** spore cluster formed by *Rhizophagus intraradices* maintained by entangled hypha. **b)** spore cluster (2—10 spores) formed by *Funnneliformis mosseae* maintained by a peridium. **c)** sporocarps of *Sclerocystis sinuosa*. **d)** central plexus of hypha from which spore develop in *Sclerocystis sinuosa*. **e)** large sporocarps of *Diversispora epigaea*. **f)** spore of *Redeckera megalocarpa* showing the straight septum formed at the base of the spore. **g)** hyphal mantle formed by sporocarps of *Sclerocystis sinuosa.* **h)** highly irregular spore shape with laminated layer reacting on Melzer’s reagent in *Rhizophagus irregularis*

- 1 a. Spores with a SW only (Fig. 4a, c, d) ______________________________________________________________________________ 11
- b. Spores with a SW and one GW (Fig. 4b) ______________________________________________________________________________ 2
- 2 a. Spore diameter ranging from 29 to 95 µm (mean spore diameter < 90 µm) ___________________________________________________ 3
- b. Spore diameter ranging from 75 to 230 µm (mean spore diameter > 90 µm) __________________________________________________ 5
- 3 a. SW smooth, spores hyaline, spore diameter (29) 39 (49) µm _________________________________________________ *Paraglomus turpe*
- b. SW ornamented, spores yellow brown to brown or white to yellowish white __________________________________________________ 4
- 4 a. Spores yellow brown to brown, ornamented with pentagonal pits, L2 of SW (2.5) 3.5 (4.5) µm thick ____________ *Paraglomus bolivianum*
- b. Spores white to yellowish white, with round shallow inconspicuous pits, L2 of SW (2.0) 2.4 (2.6) µm thick __ *Paraglomus pernambucanum*
- 5 a. SW not ornamented (smooth) _______________________________________________________________________________________ 6
- b. SW ornamented __________________________________________________________________________________________________ 7
- 6 a. Spores rusty brown to dark reddish brown, L2 of SW (2.5) 6.5 (7.5) µm thick __________________________________ *Pacispora robigina*
- b. Spores hyaline to shiny bright white, L2 of SW (4.0) 5.2 (6.5) µm thick ____________________________________ *Pacispora franscicana*
- 7 a. SW formed by 2 layers ____________________________________________________________________________________________ 8
- b. SW formed by 3 layers ___________________________________________________________________________________________ 10
- 8 a. Spore ornamented with warts, GW formed by 2 layers ___________________________________________________________________ 9
- b. Spore ornamented with dome-shaped or bottle-shaped structures, GW formed by 1 layer _______________________ *Pacispora patagonica*
- 9 a. L1 of SW laminated, 3.5 to 4.5 µm thick, L2 of GW stains pinkish red in Melzer ________________________ *Pacispora chimonobambusae*
- b. L2 of SW laminated, (2.0) 3.5 (4.5) µm thick, L2 of GW stains dull red to deep red in Melzer ____________________ *Pacispora dominikii*
- 10 a. Spore diameter (110) 122—150 (165) µm, L2 of SW 3.8—9.0 µm thick ______________________________________ *Pacispora coralloidea*
- b. Spore diameter 180—230 µm, L2 of SW (1.0) 2.0 (3.0) µm thick ___________________________________________ *Pacispora scintillans*
- 11 a. SW ornamented ________________________________________________________________________________________________ 12
- b. SW not ornamented (smooth) _____________________________________________________________________________________ 23
- 12 a. Spore diameter ranging from 43 to 140 µm, with mean spore diameter < 100 µm ____________________________________________ 13
- b. Spore size ranging from 75 to 280 µm, with mean spore diameter > 100 µm ________________________________________________ 16
- 13 a. Spores subhyaline to cream, with labyrinthiform ornamentation, L2 of SW (0.6) 1.0 (1.3) µm thick ____________ *Paraglomus brasilianum*
- b. Spores pale yellow to yellowish to yellow brown to orange brown ________________________________________________________ 14
- 14 a. Spores ornamented with cones and circles, SW formed by 4 layers _________________________________________ *Diversispora conica*
- b. Spores ornamented with pits and pustules, SW formed by 2 or 3 layers ____________________________________________________ 15
- 15 a. Spores yellow white to golden yellow, SW formed by 2 layers, L2 of SW (3.2) 4.4 (6.1) µm thick with pitted inner surface ________________________________________________________________________________________________________ *Diversispora insculpta*
- b. Spores pale yellow to yellow brown or orange brown, SW formed by 3 layers, L2 of SW (3.0) 6.5 (10.0) µm thick with L1 ornamented with blistery cup or irregularly shaped outgrowths __________________________________________ *Diversispora pustulata*
- 16 a. Spores ornamented with blister-like swellings in the L1 of SW, spores pale to grayish yellow ______________ *Diversispora slowinskiensis*
- b. Spores ornamented with spines, warts or pits in the L2 or L3 of SW, spores yellow to deep yellow, to brown to dark orange brown ____ 17
- 17 a. Ornamentation present only on the L2 of SW _________________________________________________________________________ 18
- b. Ornamentation present only on the L3 of SW _________________________________________________________________________ 20
- 18 a. Spores ornamented with warts, spore diameter (100) 138 (200) µm, L1 of SW (3.0) 5.0 (15.0) µm thick, swelling in lactic acid or PVLG into columns that radiate up to 100 µm ___________________________________________________ *Halonatospora pansihalos* (Fig. 4f)
- b. Spores ornamented with spines, L1 not expanding in lactic acid or PVLG __________________________________________________ 19
- 19 a. Spore diameter (140) 235 (330) µm, L2 of SW (4.0) 7.0 (10.0) µm thick _______________________________ *Funneliformis monosporus*
- b. Spore diameter (200) 240 (280) µm, L2 of SW (10.0) 12.5 (15.0) µm thick _______________________________ *Funneliformis halonatus*
- 20 a. L3 of SW ornamented with warts projecting inwards ___________________________________________________________________ 21
- b. L3 of SW ornamented with pits ___________________________________________________________________________________ 22
- 21 a. Spores yellow, L2 of SW (4.3) 5.6 (6.8) µm thick, L3 of SW (1.6) 1.9 (2.2) µm thick, warts 0.2—0.3 µm wide __ *Funneliformis kerguelensis*
- b. Spores bright yellowish orange to dark brownish orange, L2 of SW (5.1) 9.8 (12.5) µm thick, L3 of SW (0.6) 1.1 (1.5) µm thick, warts 0.5—0.7 µm wide ______________________________________________________________________ *Funneliformis verruculosum*
- 22 a. Spores deep yellow to brown, (170) 215 (230) µm diameter, L2 of SW ornamented with ingrowths filling pits of L3 ______________________________________________________________________________________________________________ *Funneliformis multiforus*
- b. Spores orange brown to dark orange brown, (90) 120 (151) µm diameter, only L3 of SW ornamented with pits ______________________________________________________________________________________________________________ *Funneliglomus sanmartinensis*
- 23 a. SW consisting of 5 or 6 layers ____________________________________________________________________________________ 24
- b. SW consisting of 1, 2, 3 or 4 layers ________________________________________________________________________________ 27
- 24 a. SW with 6 layers, spores pastel yellow, spore diameter (130) 173 (230) µm ___________________________ *Complexispora multistratosa*
- b. SW with 5 layers ______________________________________________________________________________________________ 25
- 25 a. Spore diameter > 150 µm, L2 of SW stains in Melzer, funneliform subtending hypha ________________________ *Funneliformis caesaris*
- b. Spore diameter < 150 µm, L2 of SW not staining in Melzer, cylindrical subtending hypha _____________________________________ 26
- 26 a. Spores formed singly or in sporocarps, hyaline to light yellow, L4 of SW 0.5 µm thick ________________________ *Diversispora gibbosa*
- b. Spores formed singly only, yellowish white to yellow brown, L4 of SW (2.4) 3.6 (5.0) µm thick ______________ *Entrophospora glacialis*
- 27 a. SW with only 1 layer ____________________________________________________________________________________________ 28
- b. SW with 2, 3 or 4 layers _________________________________________________________________________________________ 37
- 28 a. Spores white, pale yellow to light yellow, pale tan to ochre ______________________________________________________________ 29
- b. Spores yellow brown to brown, dark brown to reddish brown ____________________________________________________________ 34
- 29 a. Spore diameter 140 to 240 µm (mean > 150 µm) ______________________________________________________________________ 30
- b. Spore diameter 38 to 125 µm (mean < 150 µm) _______________________________________________________________________ 31
- 30 a. Spores pale yellow to light yellow, L1 of SW (1.2) 2.2 (2.6) µm thick, spores individually covered by hyphal mantle (Fig. 4g) _________________________________________________________________________________________________ *Sieverdingia tortuosa* (Fig. 4g)
- b. Spores opaque to milky white, L1 of SW (3.0) 4.0 (5.0) µm thick, spores not individually covered by hyphal mantle _________________________________________________________________________________________________________________ *Paraglomus lacteum*
- 31 a. Spores pale to light yellow _______________________________________________________________________________________ 32
- b. Spores pale tan to ochre _________________________________________________________________________________________ 33
- 32 a. Spores formed in loose clusters, spores ovoid to ellipsoid, mean spore diameter 67 µm ________________________ *Redeckera canadensis*
- b. Spores formed in compact sporocarps, spores oblong, elliptical to oval, subpyriform, mean spore diameter 90 µm _______ *Redeckera fulva*
- 33 a. Spores obovate to pyriform, spore diameter (38) 76 (115) µm, SW (1.7) 2.8 (4.0) µm thick ____________ *Redeckera megalocarpa* (Fig. 7f)

- b. Spores globose to subglobose, spore diameter (60) 67 (85) µm, SW (3.0) 4.0 (5.0) µm thick _____________________ *Redeckera pulvinata*
- 34 a. Spore diameter 80 to 185 µm (mean > 140 µm), clavate to subglobose, SW 17—25 µm thick at the apex __________ *Sclerocystis clavispora*
- b. Spore diameter 27 to 150 µm (mean < 140 µm), mostly obovoid, ellipsoid, fusiform elliptical to clavate, SW 7.5—25 µm thick at the apex ______________________________________________________________________________________________________ 35
- 35 a. Spore diameter 35 to 102 µm (mean < 70 µm), SW 2.0—2.5 µm (mean = 2.3 µm) thick, SW 4.0 µm thick at the spore base _________________________________________________________________________________________________________ *Sclerocystis coremioides*
- b. Spore diameter 27 to 150 µm (mean > 70 µm), SW 1.3—5.0 µm (mean > 2.3 µm) thick, SW > 4.0 µm thick at the spore base _________ 36
- 36 a. Spore diameter (27) 80 (133) µm, SW 2.0—5.0 (mean = 3.5) µm thick, SW 7.5—25 µm thick at spore apex ______ *Sclerocystis liquidambaris*
- b. Spore diameter (30) 90 (150) µm, SW 1.3—4.9 (mean = 4.1) µm thick, SW 10.0 µm thick at spore apex _____________ *Sclerocystis sinuosa* (Fig. 7c, d, g)
- 37 a. SW with 2 layers (Fig. 4a) ________________________________________________________________________________________ 38
- b. SW with 3 (Fig. 4c, d, e) or 4 layers ______________________________________________________________________________ 86
- 38 a. Spore color hyaline, subhyaline, white, pale ivory _____________________________________________________________________ 39
- b. Spore color in shades of yellow, or orange, or brown, or black ___________________________________________________________ 57
- 39 a. Spore diameter ranging from 40 to 260 µm and mean spore diameter > 70 µm ______________________________________________ 40
- b. Spore diameter ranging from 10 to 97 µm and mean spore diameter < 70 µm _______________________________________________ 45
- 40 a. SW staining in Melzer ___________________________________________________________________________________________ 41
- b. SW not staining in Melzer ________________________________________________________________________________________ 43
- 41 a. L2 of SW > 3.0 µm thick _________________________________________________________________________________________ 42
- b. L2 of SW (0.5) 1.2 (2.0) µm thick, L1 and L2 of SW stain dull orange to yellow ______________________________ *Paraglomus albidum*
- 42 a. L2 of SW (3.7) 6.1 (9.4) µm thick, L1 of SW stains pale pink in Melzer __________________________________ *Entrophospora candida*
- b. L2 of SW (4.0) 6.0 (8.0) µm thick, L1 of SW stains pale pink to orange red ______________________________ *Ambispora* spp. (glomoid morphotype – need acaulosporoid morphotype to identify species)
- 43 a. Spores hyaline, L2 of SW (5.9) 7.2 (7.9) µm thick _____________________________________________________ *Paraglomus laccatum*
- b. Spores pale ivory, subhyaline to pale yellow, L2 of SW ranging from 1.2 to 4.2 µm thick (mean < 3.0 µm) ________________________ 44
- 44 a. Spores pale ivory, mostly tear-drop to irregular in shape, L2 of SW (1.2) 2.3 (3.8) µm thick ____________________ *Diversispora eburnea*
- b. Spores subhyaline, mostly globose to subglobose in shape, L2 of SW (1.4) 2.8 (4.2) µm thick ____________________ *Diversispora spurca*
- 45 a. Maximum spore diameter 29—45 µm, and mean spore diameter ≤ 30 µm ___________________________________________________ 46
- b. Maximum spore diameter 50—97 µm, and mean spore diameter > 30 µm ___________________________________________________ 51
- 46 a. L2 of SW ranging from 0.7 to 4.0 µm thick and average thickness of L2 > 1.0 µm ___________________________________________ 47
- b. L2 of SW ranging from 0.8 to 1.2 µm thick and average thickness of L2 ≤ 1.0 µm ___________________________________________ 50
- 47 a. Maximum thickness of L2 of SW > 2.0 µm __________________________________________________________________________ 48
- b. Maximum thickness of L2 of SW < 2.0 µm __________________________________________________________________________ 49
- 48 a. L1 of SW stains in Melzer, spore diameter (12) 28 (35) µm, L2 of SW (0.8) 1.4 (2.2) µm thick __________________ *Dominikia bonfantae*
- b. L2 of SW stains in Melzer, spore diameter (20) 30 (45) µm, L2 of SW (1.1) 2.1 (3.0) µm thick ________________ *Nanoglomus plukenetia*
- 49 a. L1 of SW (1.0) 1.4 (1.7) µm thick, L2 of SW (1.0) 1.4 (1.7) µm thick _________________________________ *Microkamienskia perpusilla*
- b. L1 of SW (0.5) 0.8 (1.1) µm thick, L2 of SW (0.7) 1.1 (1.1) µm thick _________________________________ *Microkamienskia peruviana*
- 50 a. Spore diameter (11) 25 (35) µm, L1 of SW 0.8 to 1.2 µm thick, L2 of SW 0.8 to 1.2 µm thick _________________ *Microdominikia litorea*
- b. Spore diameter (10) 22 (39) µm, L1 of SW 0.6 to 1.2 µm thick, L2 of SW 0.8 to 1.2 µm thick _____________ *Microkamienskia divaricata*
- 51 a. Minimum spore diameter 17—20 µm, maximum spore diameter 50—65 µm, average spore diameter > 30 and < 45 µm ________________ 52
- b. Minimum spore diameter 17—44 µm, maximum spore diameter 58—97 µm, average spore diameter > 45 and < 60 µm ________________ 55
- 52 a. L1 of SW 0.2—0.7 µm thick (mean < 0.8 µm) _____________________________________________________________ *Dominikia minuta*
- b. L1 of SW 0.6—2.3 µm thick (mean > 0.8 µm) _________________________________________________________________________ 53
- 53 a. Spores formed singly, L2 of SW (3.8) 5.1 (7.3) µm ______________________________________________________* Pervetustus simplex*
- b. Spores formed in sporocarps ______________________________________________________________________________________ 54
- 54 a. Spores generally globose to subglobose, or ovoid, or prolate to irregular, L2 of SW (1.2) 1.8 (4.0) µm thick ________ *Delicatispora indica*
- b. Spores globose to subglobose only, L2 of SW (0.7) 1.0 (1.5) µm thick ______________________________________ *Kamienskia bistrata*
- 55 a. Spores formed in epigeous or subhypogeous sporocarps, L2 of SW (4.8) 6.5 (9.0) µm thick ________________ *Sclerocarpum amazonicum*
- b. Spores formed in loose clusters, L2 of SW ranging from 1.0 to 3.0 µm thick (mean < 2.5 µm) __________________________________ 56
- 56 a. Spores hyaline, globose to ovoid to oblong, L2 of SW (1.1) 1.6 (2.3) µm thick _________________________ *Microviscospora peruviscosa*
- b. Spores hyaline to white, globose to subglobose, L2 of SW (1.0) 2.0 (3.0) µm thick _____________________________ *Viscospora viscosa*
- 57 a. Spores with shades of yellow (pale, olive, deep, golden, bright) __________________________________________________________ 58
- b. Spores with shades of orange to brown ______________________________________________________________________________ 75
- 58 a. Spores generally globose to subglobose _____________________________________________________________________________ 59
- b. Spores generally clavate, cylindroclavate, triangular to irregular, L2 of SW (1.5) 3.2 (5.0) µm thick ____________ *Sclerocystis taiwanensis*
- 59 a. Maximum spore diameter > 110 µm and average spore diameter > 85 µm __________________________________________________ 60
- b. Maximum spore diameter ≤ 110 µm and average spore diameter < 85 µm __________________________________________________ 67
- 60 a. Average spore diameter 102 to 150 µm _____________________________________________________________________________ 61
- b. Average spore diameter 85 to 90 µm _______________________________________________________________________________ 66
- 61 a. SW staining in Melzer’s reagent ___________________________________________________________________________________ 62
- b. SW not staining in Melzer’s reagent ________________________________________________________________________________ 64
- 62 a. Spores formed mostly in compact sporocarps, L2 of SW (4.0) 6.5 (9.0) µm thick, only L2 stains in Melzer _________ *Diversispora epigaea* (Fig. 7e)
- b. Spores formed only singly in the soil, not in sporocarps, L1 or L1 and L2 stains in Melzer _____________________________________ 63
- 63 a. L1 stains pink to reddish purple in Melzer, L2 of SW (4.4) 5.2 (6.4) µm thick _____________________________ *Entrophospora etunicata*
- b. L1 and L2 stain yellow in Melzer, L2 of SW (7.5) 9.5 (10.0) µm thick ________________________________ *Diversispora przelewicensis*
- 64 a. Spores formed singly in the soil, pale to dark yellow, L2 of SW (2.6) 3.3 (4.4) µm thick ______________________ *Diversispora vistulana*
- b. Spores formed only in sporocarps __________________________________________________________________________________ 65
- 65 a. L1 of SW swelling in lactic acid, spore diameter (70) 115 (140) µm, L2 of SW (6.0) 9.0 (12.0) µm thick __ *Glomus macrocarpum* (Fig. 4a)
- b. L2 of SW not swelling in lactic acid, spore diameter (120) 150 (180) µm, L2 of SW (5.5) 6.3 (7.0) µm thick ____ *Diversispora versiformis*
- 66 a. L2 of SW stains red in Melzer, L2 of SW (2.5) 7.5 (12.4) µm thick ___________________________________________ *Glomus ibericum*
- b. L2 of SW not staining in Melzer, L2 of SW (1.0) 1.7 (2.7) µm thick _____________________________________ *Septoglomus africanum*
- 67 a. Average spore diameter > 70 µm __________________________________________________________________________________ 68
- b. Average spore diameter < 70 µm __________________________________________________________________________________ 71
- 68 a. Spores not formed in sporocarps, only singly in the soil ________________________________________________________________ 69
- b. Spores formed in sporocarps (loose clusters) and singly in the soil ________________________________________________________ 70
- 69 a. L1 of SW stains pinkish white to orange red in Melzer, L2 of SW (3.3) 7.3 (11.8) µm thick ___________________ *Entrophospora hanlinii*
- b. L1 of SW not staining in Melzer, L2 of SW (3.3) 6.2 (7.5) µm thick ____________________________________ *Diversispora varaderana*
- 70 a. Spores yellowish white to maize yellow, L2 of SW (3.3) 5.1 (7.5) µm thick _________________________________*Diversispora peridiata*
- b. Spores pale yellow to brownish yellow, L2 of SW (2.2) 2.8 (3.3) µm thick _____________________________* Macrodominikia compressa*
- 71 a. Layers of SW not staining in Melzer ________________________________________________________________________________ 72
- b. Layers of SW staining in Melzer ___________________________________________________________________________________ 73
- 72 a. Spores deep yellow to orange yellow, L1 of SW (1.2) 2.5 (3.6) µm thick and L2 of SW (3.8) 5.2 (7.5) µm thick ____ *Diversispora sabulosa*
- b. Spores pale yellow, L1 of SW (0.5) 0.6 (0.7) µm thick and L2 of SW (2.0) 2.2 (2.5) µm thick ______________________*Redeckera fragilis*
- 73 a. L1 stains pinkish white to dark red and L2 stains pale orange to pale red in Melzer, laminae of L2 pliable easily separating from each other _____________________________________________________________________________________ *Rhizophagus arabicus*
- b. Only L1 staining in Melzer, laminae of L2 not pliable __________________________________________________________________ 74
- 74 a. Spore diameter (19) 33 (52) µm, L2 of SW (1.8) 2.2 (2.5) µm thick, L1 of SW stains pinkish to purple in Melzer ____ *Dominikia bernensis*
- b. Spore diameter (39) 59 (98) µm, L2 of SW (1.3) 2.4 (5.0) µm thick, L1 of SW stains purplish pink to deep magenta in Melzer ___________ ___________________________________________________________________________________________ *Septoglomus jasnowskae*
- 75 a. Minimum spore diameter 110—175 µm, maximum spore diameter 200—340 µm, and average spore diameter > 170 µm _______________ 76
- b. Minimum spore diameter 20—60 µm, maximum spore diameter 65—140 µm, and average spore diameter < 170 µm __________________ 79
- 76 a. Spores formed singly in the soil, spores dark red brown to reddish black to almost black ______________________________________ 77
- b. Spores formed in sporocarps, spores yellow orange to orange to red brown to dark brown _____________________________________ 78
- 77 a. Spores ovoid to ellipsoid, rarely globose to subglobose, spore diameter (125) 173 (208) µm, L2 of SW (4.0) 6.0 (8.0) µm thick ___________________________________________________________________________________________________ *Septoglomus altomontanum*
- b. Spores globose to subglobose, spore diameter (150) 240 (330) µm, L2 of SW (5.0) 7.5 (9.0) µm thick _________ *Septoglomus constrictum*
- 78 a. L2 of SW (6.1) 9.7 (13.2) µm thick, L1 not swelling in acidic mountants ___________________________________ *Funneliformis pilosus*
- b. L2 of SW (2.3) 4.7 (6.9) µm thick, L1 swells in acidic mountants into flame-like or radiating columns _________ *Funneliformis coronatum*
- 79 a. Maximum spore diameter 120 to 150 µm and mean spore diameter > 70 and < 170 µm _______________________________________ 80
- b. Maximum spore diameter 65 to 90 µm and mean spore diameter > 45 and < 70 µm __________________________________________ 83
- 80 a. Spores formed in compact sporocarps (Fig. 7c, e) ____________________________________________________________________ 81
- b. Spores not formed in compact sporocarps ___________________________________________________________________________ 82
- 81 a. Spores obovoid, ellipsoid, L1 of SW (0.5) 1.8 (3.0) µm thick and L2 of SW (3.0) 6.8 (7.6) µm thick _____________ *Sclerocystis rubiformis*
- b. Spores globose to pyriform, L1 of SW 1.0 µm thick and L2 of SW 7.0 µm thick _______________________________ *Diversispora tenera*
- 82 a. Spore wall layers not staining in Melzer, L2 of SW 1.0 µm thick ________________________ *Funneliformis dimorphicum* (morph 2—M2)
- b. L2 of SW stains dark red brown in Melzer, L2 of SW (4.0) 5.0 (6.0) µm thick ____________________________ *Blaszkowskia deserticola*
- 83 a. Spores always formed in sporocarps (compact or loose clusters) __________________________________________________________ 84
- b. Spores formed in sporocarps and singly in the soil _____________________________________________________________________ 85
- 84 a. Spores formed in compact sporocarps, spores subglobose to ovoid, rarely globose, L2 of SW (1.5) 3.0 (4.0) µm thick ____ *Dominikia aurea*
- b. Spores formed in loose clusters, spores globose, L2 of SW (3.0) 4.5 (6.0) µm thick ________________________ *Rhizophagus invermaium*
- 85 a. Spores brownish orange to dark brown, L2 of SW (2.0) 3.8 (7.0) µm thick ___________________________________ *Septoglomus fuscum*
- b. Spores pale orange to brown, L2 of SW (1.0) 1.7 (3.5) µm thick ________________________________________ *Septoglomus nakheelum*
- 86 a. SW with 3 layers _______________________________________________________________________________________________ 87
- b. SW with 4 layers ______________________________________________________________________________________________ 145
- 87 a. Spores formed exclusively in sporocarps ____________________________________________________________________________ 88
- b. Spores formed exclusively singly in the soil or in sporocarps and singly in the soil __________________________________________ 104
- 88 a. Sporocarps compact epigeous _____________________________________________________________________________________ 89
- b. Sporocarps loose to compact hypogeous ____________________________________________________________________________ 92
- 89 a. Spore diameter (64) 72 (80) µm and L2 of SW (1.6) 2.0 (2.4) µm thick _____________________________________ *Redeckera avelingiae*
- b. Spore diameter (30) 38—40 (46) µm and L2 of SW (1.8) 6.5—12.5 (10.5) µm thick ____________________________________________ 90
- 90 a. Spores pale gray to pale yellow, L1 of SW 0.1—1.3 µm thick (mean = 1.0 µm), L2 of SW 5.2—8.0 µm thick (mean = 6.5 µm), L2 and L3 stains in Melzer __________________________________________________________________________ *Epigeocarpum japonicum*
- b. Spores hyaline to light yellow, L1 of SW 0.8—2.0 µm thick (mean > 1.0 µm), L2 of SW 1.8—10.5 (mean > 6.5 µm), only L2 stains in Melzer ______________________________________________________________________________________________________ 91
- 91 a. L2 of SW (1.8) 8.2 (10.5) µm thick, sporocarps 3.3 × 3.4 mm, L2 stains pastel red to brownish violet in Melzer _______________________________________________________________________________________________________________ *Dominikia glomerocarpica*
- b. L2 of SW (2.0) 8.0 (12.5) µm thick, sporocarps 1.65 × 1.69 mm, L2 stains reddish white to brownish violet in Melzer ____________________________________________________________________________________________________________ *Epigeocarpum crypticum*
- 92 a. Spore color hyaline or hyaline to light/pastel yellow ___________________________________________________________________ 93
- b. Spore color with shades of yellow to orange or brown __________________________________________________________________ 97
- 93 a. L3 of SW 0.5—1.0 µm thick with mean < 1.0 µm ______________________________________________________________________ 94
- b. L3 of SW 1.2—3.6 µm thick with mean > 1.0 µm ______________________________________________________________________ 96
- 94 a. L2 of SW 1.0—2.0 µm thick (mean < 2.0 µm), layers of SW not staining in Melzer __________________________ *Dominikia paraminuta*
- b. L2 of SW 1.3—3.9 µm thick (mean > 2.0 µm), layers of SW staining in Melzer _____________________________________________ 95
- 95 a. L2 of SW (1.5) 2.5 (3.9) µm thick, L2 easily stratifying into sublayers in crushed spores ___________________________ *Dominikia achra*
- b. L2 of SW (1.3) 2.2 (2.8) µm thick, L2 sublayers tightly adherent to each other ________________________________ *Dominikia disticha*
- 96 a. L1 of SW stains pastel red to brownish red in Melzer, L3 of SW (1.2) 2.0 (2.6) µm thick not staining in Melzer _______ *Dominikia iranica*
- b. L1 of SW stains orange white in Melzer, L3 of SW (1.6) 2.4 (3.6) µm thick staining grayish red in Melzer _________ *Dominikia lithuanica*
- 97 a. Spore color with shades of yellow (light/pastel/pale/grayish) ____________________________________________________________ 98
- b. Spore color with shades of orange or brown _________________________________________________________________________ 101
- 98 a. Spores formed in loose to compact clusters, spore diameter ranging from 20 to 86 µm (mean < 60 µm) ___________________________ 99
- b. Spores formed only in loose clusters (Fig. 7a), spore diameter ranging from 38 to 110 µm (mean > 60 µm) _______________________ 100
- 99 a. Spores light yellow to orange yellow, L2 of SW (1.6) 2.2 (3.4) µm thick, L1 stains royal purple to pinkish purple in Melzer __________________________________________________________________________________________________________ *Dominikia gansuensis*
- b. Spores pale yellow to yellow brown, L2 of SW < 0.5 µm thick, L1 stains pale to dull red and L3 stains pale to brownish red in Melzer _________________________________________________________________________________________________ *Dominikia duoreactiva*
- 100 a. Spores pastel yellow to pale yellow, sporocarps with few to more than 100 spores, L2 of SW (1.8) 2.7 (4.0) µm thick ____ *Glomus bareae*
- b. Spores grayish orange to yellowish brown, sporocarps with 5—20 spores, L2 of SW (1.0) 1.5 (2.0) µm thick ________ *Glomus mongioiense*
- 101 a. L3 of SW ranging from 2.8 to 9.8 µm thick (mean > 2.0 µm) __________________________________________________________ 102
- b. L3 of SW ranging from 0.5 to 2.0 µm thick (mean < 2.0 µm) __________________________________________________________ 103
- 102 a. Spores grayish orange to yellowish brown, L2 of SW (1.0) 1.8 (2.5) µm thick _________________________________*Glomus atlanticum*
- b. Spores orange to brownish orange, L2 of SW (2.3) 3.1 (5.0) µm thick ___________________________________ *Orientoglomus emiratia*
- 103 a. Spores orange to light brown formed in loose clusters, L2 stains brownish red to brownish violet in Melzer, spore diameter (86) 100 (121) µm ______________________________________________________________________________ *Rhizophagus dalpeae*
- b. Spores reddish brown to dark reddish brown formed in compact clusters, L2 not staining in Melzer, spore diameter (33) 63 (120) µm _______________________________________________________________________________ *Parvocarpum badium*
- 104 a. Spore color hyaline to subhyaline to white to pale cream and pale yellow ________________________________________________ 105
- b. Spore color yellow, or cream, or orange, or brown, or black ___________________________________________________________ 116
- 105 a. Layers of spore wall not staining in Melzer ________________________________________________________________________ 106
- b. Layers of spore wall staining in Melzer ___________________________________________________________________________ 108
- 106 a. Spores pale yellow, spore diameter (104) 170 (246) µm _______________________________________________ *Funneliformis huaxica*
- b. Spores hyaline, spore diameter 31 to 78 µm (mean < 70 µm) __________________________________________________________ 107
- 107 a. L1 of SW 1.1—2.8 µm thick (mean = 1.8 µm) and L2 of SW 0.8—1.6 µm thick (mean = 1.2 µm) ______________ *Dominikia difficilevidera*
- b. L1 of SW 0.5—1.0 µm thick (mean = 0.7 µm) and L2 of SW 2.5—4.4 µm thick (mean = 3.5 µm) __________________ *Innospora majewski*
- 108 a. Spores with only 1 layer (L1 or L3) of SW staining in Melzer ________________________________________________________ 109
- b. Spores with 2 layers of SW staining in Melzer ____________________________________________________________________ 114
- 109 a. Spores with L1 staining in Melzer _______________________________________________________________________________ 110
- b. Spores with L3 staining in Melzer _______________________________________________________________________________ 113
- 110 a. L2 of SW 3.5 to 14.5 µm thick (mean > 5.0 µm) ____________________________________________________________________ 111
- b. L2 of SW 2.0 to 6.4 µm thick (mean < 5.0 µm) _____________________________________________________________________ 112
- 111 a. Spore diameter (100) 180 (260) µm, L2 of SW (9.0) 11.3 (14.5) µm thick, L2 of granular consistency causing cracking _____________________________________________________________________________________________________________ *Rhizophagus clarus*
- b. Spore diameter (55) 81 (95) µm, L2 of SW (3.5) 5.5 (9.5) µm thick, L2 not of granular consistency ____________ *Entrophospora walkeri*
- 112 a. Spores hyaline to white, L1 of SW stains light pink in Melzer, spores formed only singly in the soil _________________ *Oehlia diaphana*
- b. Spores ivory to pinkish cream, L1 of SW stains orange in Melzer, spores formed singly in the soil and in loose clusters _____________________________________________________________________________________________________________ *Diversispora celata*
- 113 a. L2 of SW (6.0) 9.0 (12.0) µm thick, L3 of SW < 1.0 µm thick and stains faint pink in Melzer _______________ *Entrophospora lamellosa*
- b. L2 of SW (0.6) 1.8 (5.0) µm thick, L3 of SW (1.5) 2.4 (4.4) µm thick and stains pale orange to deep red in Melzer ______________________________________________________________________________________________________ *Rhizophagus irregularis* (Fig. 7h)
- 114 a. L2 of SW (< 0.5) 0.8 (1.2) µm thick, L2 and L3 stain light yellow in Melzer ________________________ *Paraglomus occultum* (Fig. 4e)
- b. L2 of SW ranging from 2.8 to 8.8 (mean > 4.0 µm) __________________________________________________________________ 115
- 115 a. L2 of SW (4.0) 6.0 (8.8) µm thick, L1 of SW stains pinkish white to dark red and L3 stains pale yellow _________ *Rhizophagus dunensis*
- b. L2 of SW (2.8) 4.1 (5.4) µm thick, L1 and L2 of SW stain light to dark yellow ________________________________ *Diversispora clara*
- 116 a. Spore color in shades of yellow, cream, ochraceous, and straw _________________________________________________________ 117
- b. Spore color in shades of orange and brown ________________________________________________________________________ 131
- 117 a. Layers of SW not staining in Melzer ______________________________________________________________________________ 118
- b. Layers of SW staining in Melzer _________________________________________________________________________________ 123
- 118 a. Maximum spore size 200—240 µm and mean spore diameter > 100 µm ___________________________________________________ 119
- b. Maximum spore size 70—120 µm and mean spore diameter ≤ 100 µm ____________________________________________________ 120
- 119 a. Spores pale yellow to pale brownish yellow, L2 of SW (1.0) 2.0 (3.0) µm thick and L3 of SW (1.0) 8.0 (15.0) µm thick __________________________________________________________________________________________________________ *Diversispora trimurales*
- b. Spores yellow brown to dark orange brown, L2 of SW (6.0) 10.0 (14.0) µm thick and L3 of SW (1.0) 1.7 (2.5) µm thick _______________________________________________________________________________________________________ *Funneliformis geosporus*
- 120 a. L1 of SW (1.2) 2.5 (3.6) µm thick, L3 of SW (1.3) 2.5 (3.2) µm thick, spores creamy white to pale ochraceous ____ *Diversispora cerifera*
- b. L1 of SW ranging from 0.5 to 1.5 µm thick (mean < 2.5), L3 of SW ranging from 0.5 to 2.2 µm thick (mean < 2.5 µm) ____________ 121
- 121 a. Spore diameter (23) 50 (70) µm, mean thickness of L1 < 1.0 µm, spores light yellow to yellow ochre ___________ *Septoglomus xanthium*
- b. Spore diameter ranging from 45 to 120 µm (mean 95 to 98 µm), mean thickness of L1 ≥ 1.0 µm ______________________________ 122
- 122 a. Spores yellowish white to dark yellow, L2 of SW (1.0) 3.8 (7.5) µm thick ___________________________ *Entrophospora argentinensis*
- b. Spores golden yellow to deep orange, L2 of SW (1.7) 5.8 (8.8) µm thick __________________________________ *Diversispora aurantia*
- 123 a. Spores with only 1 layer of SW staining in Melzer ___________________________________________________________________ 124
- b. Spores with at least 2 layers of SW staining in Melzer ________________________________________________________________ 129
- 124 a. Spores with L1 staining in Melzer ________________________________________________________________________________ 125
- b. Spores with L2 or L3 staining in Melzer ___________________________________________________________________________ 127
- 125 a. L2 of SW (0.8) 1.2 (1.6) µm thick, spore diameter (100) 195 (260) µm, subtending hypha broadly funnel-shaped _ *Funneliformis mosseae* (Fig. 6h, 7b)
- b. L2 of SW ranging from 1.5 to 6.2 µm (mean = 3.2 to 3.8 µm), subtending hypha cylindrical to slightly flared ___________________ 126
- 126 a. Spores white to pale cream to yellow brown with a greenish tint, L3 of SW (3.2) 7.2 (12.0) µm thick _________ *Rhizophagus intraradices* (Fig. 4c, d, 7a)
- b. Spores cream to light yellow, L3 of SW < 0.5 µm thick _____________________________________________ *Entrophospora claroidea*
- 127 a. L2 stains red brown in Melzer, L2 of SW (2.0) 7.5 (14.3) µm thick ____________________________________ *Rhizophagus fasciculatus*
- b. L3 stains in Melzer ___________________________________________________________________________________________ 128
- 128 a. L3 of SW (2.4) 3.5 (4.7) µm thick, L3 stains light yellow to bright dark yellow in Melzer ______________ *Paracorymbiglomus pacificum*
- b. L3 of SW (0.5) 0.9 (1.2) µm thick, L3 stains reddish white to grayish rose in Melzer ____________________ *Entrophospora drummondi*
- 129 a. L2 stains pale yellow and L3 stains pinkish to dull red in Melzer, spore diameter (85) 136 (170) µm, L3 of SW (4.8) 8.9 (22.0) µm _________________________________________________________________________________________________ *Desertispora omaniana*
- b. L1, L2 and L3 stain in Melzer, spore diameter ranging from 63 to 108 (mean < 100 µm) ____________________________________ 130
- 130 a. L2 of SW (6.1) 9.7 (13.2) µm thick and L3 of SW (0.6) 0.8 (1.1) µm thick ___________________________________ *Rhizoglomus cacao*
- b. L2 of SW (1.0) 1.3 (2.3) µm thick and L3 of SW (10.5) 12.0 (13.0) µm thick ___________________________ *Rhizophagus vesiculiferus*
- 131 a. Spore color in shades of chestnut, coffee brown, reddish brown, black brown _____________________________________________ 132
- b. Spore color in shades of pale orange, yellow orange, orange to raw umber ________________________________________________ 136
- 132 a. L1 of SW stains pinkish to purple in Melzer, spores dark brownish black, L3 of SW (3.8) 5.5 (7.1) µm thick _______ *Septoglomus nigrum*
- b. Layers of SW not staining in Melzer _____________________________________________________________________________ 133
- 133 a. Spore diameter ranging from 61 to 167 µm and mean spore diameter < 150 µm ___________________________________________ 134
- b. Spore diameter ranging from 137 to 400 µm and mean spore diameter > 150 µm __________________________________________ 135
- 134 a. Spores dark chestnut to coffee brown, L2 of SW (5.4) 6.0 (6.5) µm thick and L3 of SW (0.8) 1.0 (1.2) µm thick ______________________________________________________________________________________________________________ *Silvaspora neocaledonica*
- b. Spores reddish brown to dark brown, L2 of SW (1.2) 1.9 (3.3) µm thick and L3 of SW (5.3) 7.6 (11.3) µm thick _________________________________________________________________________________________________________________ *Septoglomus furcatum*
- 135 a. Spores dark chestnut brown to black brown, spore diameter (137) 195 (285) µm, L3 of SW (7.2) 9.6 (12.0) µm thick ______________________________________________________________________________________________________________ *Rhizophagus melanus*
- b. Spores dark red brown, spore diameter (243) 320 (400) µm, L3 of SW (12.8) 16.0 (19.2) µm thick _______________ *Melanoglomus titan*
- 136 a. L1 stains pinkish purple and L2 stains yellow brown in Melzer, spore diameter (47) 64 (93) µm, L2 of SW (1.1) 2.4 (3.8) µm thick ______________________________________________________________________________________________________ *Glomus chinense*
- b. Layers of SW not staining in Melzer _____________________________________________________________________________ 137
- 137 a. Spore diameter ranging from 53 to 120 µm and mean spore diameter < 100 µm ___________________________________________ 138
- b. Spore diameter ranging from 50 to 300 µm and mean spore diameter > 100 µm ___________________________________________ 140
- 138 a. L2 of SW (4.8) 8.3 (11.5) µm thick, spores pale orange to light brown __________________________________ *Diversispora densissima*
- b. L2 of SW ranging from 0.7 to 1.8 (mean < 1.5 µm) __________________________________________________________________ 139
- 139 a. Spores orange to raw umber, L3 of SW (2.0) 6.1 (8.8) µm thick, SH (3.7) 4.9 (8.1) µm thick at the spore base ____ *Diversispora arenaria*
- b. Spores yellow orange, L3 of SW (2.8) 3.0 (3.2) µm thick, SH (2.7) 3.0 (3.5) µm thick at the spore base _______ *Diversispora succinacia*
- 140 a. Maximum spore diameter ranging from 270 to 300 µm and mean spore diameter > 150 µm __________________________________ 141
- b. Maximum spore diameter ranging from 145 to 220 µm and mean spore diameter < 150 µm _________________________________ 142
- 141 a. L1 of SW (0.5) 1.3 (2.0) µm thick and L2 of SW (6.0) 18.0 (30.0) µm thick ________________________ *Paracorymbiglomus globiferum*
- b. L1 of SW (2.0) 5.0 (8.0) µm thick and L2 of SW (2.0) 5.0 (8.0) µm thick ________________ *Funneliformis dimorphicus* (morph 1—M1)
- 142 a. Spores formed in loose clusters and singly in the soil ________________________________________________________________ 143
- b. Spores formed only singly in the soil _____________________________________________________________________________ 144
- 143 a. Spores light red to black with reddish highlights, L1 of SW (0.8) 3.7 (6.6) µm thick, and L2 of SW (7.4) 14.9 (22.3) µm thick __________________________________________________________________________________________________ *Septoglomus mediterraneum*
- b. Spores pastel yellow to orange, L1 of SW (0.7) 1.1 (1.7) µm thick, and L2 of SW (3.9) 7.0 (10.0) µm thick ______________________________________________________________________________________________________________ *Corymbiglomus corymbiformis*
- 144 a. Spores reddish yellow to brownish red, L1 of SW (0.8) 1.0 (1.3) µm thick, L2 of SW (4.2) 9.2 (14.5) µm thick and SH (5.0) 8.6 (13.5) µm thick at the spore base __________________________________________________________ *Diversispora jakucsiae*
- b. Spores orange to reddish brown, L1 of SW (1.3) 1.8 (2.3) µm thick, L2 of SW (6.3) 8.4 (11.3) µm thick and SW (9.0) 11.8 (14.5) µm thick at the spore base ____________________________________________________ *Diversispora peloponnesiaca*
- 145 a. Spores formed in compact epigeous sporocarps _____________________________________________________________________ 146
- b. Spores formed in loose or compact clusters, or singly in the soil ________________________________________________________ 147
- 146 a. Spore diameter (120) 136 (148) µm, L3 of SW (5.0) 7.6 (9.8) µm thick, L4 of SW (0.4) 0.5 (0.7) µm thick ___ *Diversispora sporocarpica*
- b. Spore diameter (63) 72 (82) µm, L3 of SW (4.8) 13.7 (22.3) µm thick, L4 of SW (1.0) 1.2 (1.3) µm thick __________*Rhizophagus maiae*
- 147 a. Spores hyaline to white _______________________________________________________________________________________ 148
- b. Spores with shades of yellow, or orange, or brown __________________________________________________________________ 150
- 148 a. Spores hyaline to yellowish white, L3 of SW (2.4) 6.4 (10.2) µm thick, L1 and L4 stain in Melzer ___________ *Entrophospora furrazolae*
- b. Layers of SW not staining in Melzer _____________________________________________________________________________ 149
- 149 a. Spore diameter (63) 86 (125) µm, L3 of SW (2.6) 4.1 (4.9) µm thick _________________________________________ *Diversispora alba*
- b. Spore diameter (22) 53 (76) µm, L3 of SW (0.5) 0.8 (1.0) µm thick _____________________________________ *Rhizophagus proliferus*
- 150 a. Minimum spore diameter 150—180 µm, maximum spore diameter 228—320 µm, and mean spore diameter > 200 µm _______________ 151
- b. Minimum spore diameter 23—110 µm, maximum spore diameter 77—240 µm, and mean spore diameter < 200 µm _________________ 152
- 151 a. Spore diameter (180) 260 (320) µm, L4 of SW (4.0) 5.2 (6.4) µm thick, L1 stains pale pink in Melzer _______ *Funneliformis caledonium*
- b. Spore diameter (154) 202 (228) µm, L4 of SW (0.9) 1.2 (1.4) µm thick, L1 not staining in Melzer ____________ *Septoglomus mexicanum*
- 152 a. Minimum spore diameter 23—62 µm, maximum spore diameter 77—121 µm, and mean spore diameter < 100 µm __________________ 153
- b. Minimum spore diameter 55—110 µm, maximum spore diameter 133—240 µm, and mean spore diameter > 100 and < 200 µm _______ 156
- 153 a. L1 of SW staining in Melzer ____________________________________________________________________________________ 154
- b. Layers of SW not staining in Melzer _____________________________________________________________________________ 155
- 154 a. L1 of SW (1.3) 2.4 (4.5) µm thick, L3 of SW (3.6) 8.8 (12.8) µm thick ______________________________ *Complexispora mediterranea*
- b. L2 of SW (0.6) 0.7 (0.8) µm thick, L3 of SW (0.8) 1.5 (3.0) µm thick _________________________________________ *Glomus rugosae*
- 155 a. Spores pale yellow, L2 of SW (0.8) 1.1 (1.6) µm thick __________________________________________________ *Diversispora marina*
- b. Spores orange to dark brown, L2 of SW (1.4) 2.7 (4.0) µm thick ________________________________________ *Diversispora valentina*
- 156 a. Layers of SW not staining in Melzer ______________________________________________________________________________ 157
- b. At least 1 layer of SW staining in Melzer __________________________________________________________________________ 158
- 157 a. Spores brownish orange to dark brown, spore diameter (110) 133 (165) µm, L2 of SW (2.0) 5.1 (8.8) µm thick ___ *Septoglomus turnauae*
- b. Spores pale yellow to yellowish brown, spore diameter (77) 109 (135) µm, L2 of SW (0.8) 1.8 (2.6) µm thick ____ *Diversispora aestuarii*
- 158 a. L1 or L4 stains in Melzer ______________________________________________________________________________________ 159
- b. Two or more layers staining in Melzer ____________________________________________________________________________ 161
- 159 a. L4 of SW stains reddish brown in Melzer, spore diameter (108) 146 (191) µm, mean thickness of L4 = 5.1 µm _____________________________________________________________________________________________________________ *Funneliformis fragilistratum*
- b. L1 of SW stains in Melzer ______________________________________________________________________________________ 160
- 160 a. L1 of SW stains pinkish red in Melzer, L3 of SW (2.5) 5.5 (10.0) µm thick, L4 of SW < 0.5 µm thick ____________ *Entrophospora lutea*
- b. L1 of SW stains pink to deep red, L3 of SW (5.5) 9.4 (13.3) µm thick, L4 of SW (0.8) 0.9 (1.0) µm thick _______ *Glomus tetrastratosum*
- 161 a. L4 of SW 2.5—4.3 µm thick and mean thickness of L4 3.1 to 3.2 µm _____________________________________________________ 162
- b. L4 of SW 0.6—2.0 µm thick and mean thickness of L4 1.0 to 1.3 µm _____________________________________________________ 163
- 162 a. Spore diameter (65) 111 (138) µm, L1, L2, L3 and L4 of SW stain in Melzer, wall of SH (4.6) 5.0 (5.5) µm thick at the spore base _________________________________________________________________________________________________ *Rhizophagus silesianus*
- b. Spore diameter (110) 148 (172) µm, only L1 and L4 stain in Melzer, wall of SH (3.2) 4.0 (4.8) µm thick at the spore base __________________________________________________________________________________________________________ *Rhizophagus custos*
- 163 a. Spores pastel yellow to light yellow, L3 of SW (6.3) 8.7 (14.0) µm thick _________________________________ *Rhizophagus natalensis*
- b. Spores golden yellow brown to yellow brown, L3 of SW (2.0) 2.7 (3.5) µm thick _________________________ *Rhizoglomus venetianum*

## Discussion

We emphasized herein the role of spore morphology to identify species of AMF according to the mode of spore formation (narrowing to some extent candidates at the family and genus levels) and spore wall phenotypes (species level) interpreted within the framework of a spore developmental model. Morphological identification of an AMF species provides the first meaningful step in understanding the biology and phylogeny of a specimen. Phylogenetic analyses defining monophyletic groups via comparative morphology (homologies) (Morton 1990), trait analyses (Chaudhary et al. 2025) and molecular cladogenesis (Krüger et al. 2012) can follow. In this paper, we have created a set of keys that are strictly operational for that purpose by focusing first on mode of spore formation and then on specific traits of spores. The keys do not separate groups hierarchically according to family or genus, because the same mode of spore formation occur across different phylogenetic groups (see Table 1). Despite genetic markers having been proposed for systematics and phylotaxonomy (Lee et al. 2008; Krüger et al. 2012; Stockinger et al. 2014; Magurno et al. 2019) and environmental identification of AMF (Öpik et al. 2010; Delavaux et al. 2021, 2022), spore morphology still represents a non-expensive and practical method to identify AMF species given some basic training in morphological taxonomy. This approach has been used in different laboratories around the world, especially for field-based surveys, to generate information on AMF species distribution and community studies, and represents an essential first step in initiation of AMF single species culture.

Spores of AM fungi are the largest spores produced in the kingdom Fungi (Aguilar-Trigueros et al. 2019) and a spore trait database incorporating phenotypic characters has been proposed to improve our understanding on biology of these fungi (Chaudhary et al. 2025). AMF spores can be extracted from field soils, litter, or from pot cultures and then subjected to microscopic study under a stereoscope or compound microscope and they represent the only part of the fungal organism that differentiate phenotypes to group distinct geographical populations into species in Glomeromycota (Morton 1993). One caveat of this process is that mode of spore formation can be somehow difficult to determine under a stereoscope if precursor structures (subtending hypha, suspensor cell) are absent. In this case, the investigator should mount the spores in slides and try to determine the mode of spore formation on a compound microscope. Another caveat is that some layers forming the spore wall might not be present in mature spores, or they may be altered by parasitism, especially in field-collected spores. This is a particularly important consideration for glomoid species whose spore walls are formed by 2—3 evanescent layers that are diagnostic and tend to slough off with aging.

Morphological identification of AMF species emphasizes phenotypic variation, which may be important in revealing phenomena like epistasis (gene interactions), nongenetic inheritance, niche construction and unit of selection (Marin and Wade 2025), although the specific relationships between spore wall traits and these processes remain largely unknown. A combination of morphology and molecular methods (polyphasic approach), using available genetic markers (Öpik et al. 2010; Krüger et al. 2012; Delavaux et al. 2021, 2022), is important when characterizing AMF from natural communities.

However, identification of AMF based on morphology (spore-based) and molecular (DNA-based) approaches is subject to different methodological biases. Spore-based analyses start with spore extraction and morphological identification of spores from soil, which favors taxa that sporulate abundantly or produce easily identifiable spores, and are currently in their dormant life history phase. In fact, seasonal patterns of spore abundance in the field can be used to detect differences in seasonality (Pringle and Bever 2002; Hopkins and Bever 2024). This approach, however, fails to detect non-sporulating species or species that sporulate under specific environmental conditions (Stutz and Morton 1996) and relies on diagnostic spore phenotypes that can be ambiguous, shared among taxa, or even absent in mature spores (*e.g.* sloughing outer layers of spore wall), leading to misidentification or lumping of cryptic species. This approach detects only species that are present in the soil spore bank and fail to detect fungi that are active colonizing roots if spores are absent at the time of sampling (Dumbrell et al. 2010; Alguacil et al. 2015; Faggioli et al. 2019). Moreover, morphologica characterization is labor-intensive due to the methods used to extract spores from soils (Boyno et al. 2023) and difficult to scale to large sample sizes. Conversely, DNA-based methods, such as high-throughput sequencing of rRNA gene regions, can detect AMF taxa directly from roots or soil and therefore capture non-sporulating or low-sporulating species. However, these approaches also introduce biases related to primer specificity, PCR amplification efficiency, copy number variation in rRNA genes, and incomplete reference databases (Tedersoo et al. 2015; Nilsson et al. 2019). Furthermore, DNA detected in soil may derive from dormant propagules, hyphae, dead AMF, or extracellular DNA, making it difficult to distinguish active or living symbionts from residual genetic material (Nilsson et al. 2019). Moreover, common kits to extract environmental DNA relies on relatively small amounts (250 mg) of soil which can produce variable results (Hart et al. 2015). As a result, spore-based and molecular approaches often recover overlapping but non-identical AMF communities, and integrating both methods is increasingly recommended to obtain a more comprehensive assessment of AMF diversity and ecological function.

The spore development model proposed by Morton et al. (1995) also resulted in a nomenclature for spore phenotypes based on a sound biological process: ontogeny. We recommend that the nomenclature based on spore ontogenetic studies (spore wall, germinal wall with their differentiating layers, and germination structure) should be used in the description of new species in Glomeromycota, as it has been already accepted in some descriptions (Crossay et al. 2018; Niezgoda et al. 2024). This would standardize species description, allow direct comparison of characters between species, and make species descriptions easier to understand (especially for non-taxonomists). Other nomenclature has been proposed to refer to spore wall structures as outer wall, middle wall and inner wall with the argument that “in the Gigasporaceae spore germination always starts from the innermost layer of the innermost wall” (Oehl et al. 2008). However, in *Gigaspora* germination starts from a papillate layer associated with the spore wall and the shield or orb are always formed on the surface of the innermost germinal wall (therefore associated with the first layer of this wall) in species differentiating these germination structures. Moreover, this nomenclature fails to consider that only species that form germinal walls also differentiate a discrete germination structure (*e.g.* shield, orb) and it is reasonable to hypothesize that differentiation of germinal walls evolved as a physical support for the germination structure. We recognize three modes of spore formation in Glomeromycota: gigasporoid, acaulosporoid, and glomoid, following Walker et al. (2018). Other types of spore formation have been proposed (*e.g.* entrophosporoid, pacisporoid, racocetroid, dentiscutatoid, etc.) by Oehl et al. (2011). However, these mode of spore formation are redundant, not clearly defined, and they consider two distinct processes during ontogeny: spore formation and differentiation. There is also a conceptual problem as some modes of spore formation were defined for a taxon that lost its generic identity, becoming ambiguous. For example, the acaulo-palaeosporoid mode of spore formation was originally described for *Palaeospora spainii* (Oehl et al. 2014), however, this species was transferred to *Archaeospora* by Schüßler and Walker (2019). The fuscutatoid mode was associated with *Fuscutata aurea* (Mello et al. 2012), a genus that was synonymized with *Dentiscutata* by Redecker et al. (2013). As a result, both modes of spore formation are no longer associated with a valid genus, leaving the category without clear taxonomic support. Adopting only the three main types of spore formation as used herein helps taxonomy by reducing unnecessary complexity and avoiding categories tied to species taxon that may later be reclassified. Moreover, they represent the main ontogenetic patterns observed in Glomeromycota and make spore formation a useful character.

While developing the identification keys, we detected several instances of potential synonymies among the taxa. These cases suggest that some names currently treated as distinct may, in fact, refer to the same species. Within gigasporoid species, distinction of *Scutellospora dipurpurascens* and *Scutellospora calospora* resides in the presence of a thin layer in the germinal wall 1 in the latter, a character that could have been overlooked when the former species was described. Spore morphology and ornamentation patterns overlap considerably between *Dentiscutata reticulata* and *D. nigerita*, and both could not be separated in the key. Within acaulosporoid species, *Acaulospora morrowiae* and *A. rugosa* are practically indistinguishable, and spore color, diameter and thickness of spore wall layers highly overlap between *Acaulospora koreana* and *A. mellea*, and between *A. capsicula* and *A. colossica*. Within glomoid spores, identification is more challenging compared to acaulosporoid and gigasporoid species due to their morphological plasticity and lack of distinct phenotypes in the spore wall. We found that distinctions between some species are based on very subtle characters that overlap between species as between *Sclerocystis liquidambaris* and *S. sinuosa, Diversispora eburnea* and *D. spurca, Microkamienskia perpusilla* and *Microkamienskia peruviana*, *Microdominikia litorea* and *Microkamienskia divaricata*, *Microviscospora peruviscosa* and *Viscospora viscosa*. Recognizing and addressing these possible synonymies is crucial, as they can impact the accuracy of species identification. Further taxonomic review and comparison of morphological and genetic characters between each pair of species listed above is required to resolve these ambiguities and strength the taxonomic framework of Glomeromycota.

The number of germinal walls appears to be well conserved within genera in Glomeromycota and some discrepancies in the protologue of some species were reinterpreted herein for this character. In *Archaeospora*, some species are considered to have three layers in the spore wall (*e.g. A. trappei, A. schenckii* at INVAM) while others have been described with one (*e.g. A. europaea)* or two germinal walls (*e.g. A. spainiae*). Considering that a germination orb was described being formed over the innermost layer in *Archaeospora trappei* by Spain (1992), we considered species in this genus differentiating only one germinal wall. Similarly, protologues of *Ambispora reticulata* and *Ambispora granatensis* indicates the presence of two germinal walls, however we interpreted as being only one germinal wall as they are not clearly shown in the illustration and depart from the pattern found for other species in this genus (*e.g. Ambispora leptoticha* and *Ambispora gerdemannii*) as described and illustrated by Bills and Morton (2015). We included *Kuklospora spinosa* and *Fuscutata aurea* in the taxonomic keys, despite both genera were synonymized with *Acaulospora* and *Dentiscutata,* respectively. *Kuklospora colombiana* and *K. kentinensis* were transferred to *Acaulospora* by Kaonongbua et al. (2010) based on molecular phylogeny and spore developmental mode. However, *Kuklospora spinosa* was described by Cai et al. (2013) and the species have not yet been formaly transferred to *Acaulospora*. Similarly, the genus *Fuscutata* proposed by Oehl et al. (2008) was shown to have no phylogenetic support by Morton and Msiska (2010a). Species in this genus were then transferred to *Dentiscutata* by Redecker et al. (2013), but the publication of *Fuscutata aurea* was not available at the time and the species was not formally transferred to *Dentiscutata.*

In conclusion, the development and refinement of taxonomic keys for AMF are important for accurate identification based on spore morphology. We acknowledge that this method to identify species of AMF can be limited by absence or modification of spore wall layers in field-collected spores, potential morphological plasticity (including spore dimorphism), and convergent traits among species. However, we are confident that recognition of the main modes of spore formation, understanding of the spore developmental model and basic training in AMF spore morphology would be sufficient to use the taxonomic keys as a tool for identification. As molecular tools enhance our understanding of Glomeromycota phylogeny and improve species identification from environmental sequences, they serve as a valuable complement to traditional morphological methods. Moving forward, combining morphological and molecular approaches certainly strengthen the systematic framework for Glomeromycota and will be of paramount importance to increase reliability of species identification for this important group of soil fungi.

## Supplementary Information

Below is the link to the electronic supplementary material.

Supplementary file1 (XLSX 71 KB)

Supplementary file2 (DOCX 19 KB)

## Data Availability

All data supporting the findings of this study are available within the paper and its Supplementary Information. Morphological traits used to build the dichotomous keys are provided in Supplementary Material 1, along with sources used to mine the data.
