# Supplementary material for: Dichotomous keys for morphological identification of spores produced by arbuscular mycorrhizal fungi (phylum Glomeromycota) integrating the mode of spore formation and a spore developmental model
Source: Mycorrhiza. 2026 Jun 20;36(4):37. doi: 10.1007/s00572-026-01270-7 (PMC13283158; doi:10.1007/s00572-026-01270-7)
Supplement: Supplementary file 2 — Supplementary file2 (DOCX 19 KB) [file 572_2026_1270_MOESM2_ESM.docx]

**Glossary – key terms for AMF spore morphology**

**Acidic mountant**: a medium to mount a specimen on a microscope slide that contains an acid. A common acidic mountant used for AMF spores is Polyvinyl-Lactic Acid-Glycerol (PVLG).

**Beaded layer:** an outer layer of a germinal wall that is covered by granular excrescences (“beads”), commonly found in species of *Acaulospora*.

**Blastically formed spores**: spores formed by “blowing out” from the tip of a sporogeneous hypha, diagnostic of glomoid spores.

**Bulbous suspensor-like cell**: A swollen basal cell at the tip of a sporogenous hypha, diagnostic in gigasporoid spore formation.

**Central plexus of hypha:** dense, thick-walled, entangled mass of hyphae in the center of sporocarp that gives rise to the sporogenous hyphae from which the spores develop, commonly found in species of *Sclerocystis*.

**Cicatrix (scar):** a specific marking on the spore surface that indicates where it was once attached to the neck of a sporiferous saccule, diagnostic for acaulosporoid spores. Microscopically, the scar is round, ovoid, or circular mark, with diameter and edge appearance varying slightly among species.

**Germinal wall(s) (GW)**: Inner colorless flexible structure(s) of AMF spores that separate from the spore wall when broken, formed by 1–3 layers, that enclose the cytoplasm and are associated with germination.

**Germination structure**: A specialized structure formed on the surface of innermost germinal wall that initiates germ tube emergence. Types include: **germination orb** - a disc-shaped structure, and **germination shield** - a flattened, smooth or lobed structure.

**Hyphal mantle**: a dense, sheath-like structure formed by tightly packed hyphae around individual spores, spore clusters or sporocarps of AMF.

**Intercalary formed spores:** spores formed within (in the middle) of an undifferentiated hypha (*i.e.*, a hypha without obvious specialization like branching, thickening, or a suspensor-like swelling), diagnostic in glomoid spore formation.

**Layer(s) (L):** each discrete structure with its own properties that comprises the spore wall and any germinal walls within AMF spores.

**Laminated layer:** A layer consisting of a number of sublayers laid down as the spore matures.

**Mucilaginous layer**: a colorless layer of variable thickness, appearing gelatinous, often the outermost layer of a spore wall that reacts in Melzer’s reagent.

**Lumen**: The central hollow space inside the sporogeneous hypha. It may remain open or be closed by a septum (cross-wall), a plug or wall thickening.

**Melzer’s reagent reaction**: A staining test to observe the reaction of layers of the spore and germinal wall to iodine. Reactions encompass a range of colors (*e.g.*, blue, red-brown, red to deep purple, etc.).

**Ornamentation:** structural variation in texture and pattern on the outer or inner surface of AMF spores. Common ornamentations are warts (small, raised flat bumps), spines (sharp, spine-like projections), reticulum (net-like or honeycomb pattern formed by ridges), pits (crater-like depressions), tubercles (small, raised round bumps), and beads (see above).

**Pedicel**: a short stalk that branches from the saccule neck and produces a spore, especially in the genus *Ambispora*.

**Sloughing layer:** a single outer colorless mucilaginous layer (see above) of the spore wall that degrades so that it is absent on mature spores.

**Spore clusters:** grouping of spores formed together and maintained as a unit by entangled hyphae or sometimes by a peridium. Spore clusters are smaller and less organized than sporocarps.

**Spore diameter:** a measure of spore size (in µm). Values are reported as (minimum) mean (maximum). Ex.: (160) 250 (280) µm.

**Spore shape:** the three-dimensional form of a spore. These forms include globose, subglobose, ovoid, obovoid, ellipsoid, pyriform, irregular, oblong, reniform, fusiform, and clavate.

**Spore wall (SW)**: The main structural element of all spores originating from a sporogenous hypha (see below), consisting of 1–6 distinct layers that rarely separate when a spore is crushed. It forms before any additional structures develop. Microscopically, the spore wall separates readily from any germinal walls formed later internally. It provides structural and environmental protection for the spore.

**Sporiferous saccule (saccule)**: A balloon-like structure formed blastically from a sporogenous hypha, with a narrow **neck** that connects it to the parent hyphal network. Spores can form on the side of the neck, within the neck, or inside the saccule.

**Sporocarp:** a specialized, multicellular, usually macroscopic structure that contains few (3-10) to thousands of spores. Sporocarps can be **epigeous** (above ground) or **hypogeous** (underground).

**Sporogenous hypha (= subtending hypha)**: The hypha that connects the spore to the parent hyphal network.

**Sublayers:** each individual layer laid down to form a laminated layer (see above) comprising the spore wall
